# Selection of a promiscuous minimalist cAMP phosphodiesterase from a library of de novo designed proteins

**DOI:** 10.1038/s41557-024-01490-4

**Published:** 2024-05-03

**Authors:** J. David Schnettler, Michael S. Wang, Maximilian Gantz, H. Adrian Bunzel, Christina Karas, Florian Hollfelder, Michael H. Hecht

**Affiliations:** 1 Department of Biochemistry, University of Cambridge, Cambridge, UK; 2 Department of Chemistry, Princeton University, Princeton, USA; 3 Department of Biosystems Science and Engineering, ETH Zürich, Basel, Switzerland; 4 Department of Molecular Biology, Princeton University, Princeton, USA

**Keywords:** Enzymes, Enzymes, Microfluidics

## Abstract

The ability of unevolved amino acid sequences to become biological catalysts was key to the emergence of life on Earth. However, billions of years of evolution separate complex modern enzymes from their simpler early ancestors. To probe how unevolved sequences can develop new functions, we use ultrahigh-throughput droplet microfluidics to screen for phosphoesterase activity amidst a library of more than one million sequences based on a de novo designed 4-helix bundle. Characterization of hits revealed that acquisition of function involved a large jump in sequence space enriching for truncations that removed >40% of the protein chain. Biophysical characterization of a catalytically active truncated protein revealed that it dimerizes into an α-helical structure, with the gain of function accompanied by increased structural dynamics. The identified phosphodiesterase is a manganese-dependent metalloenzyme that hydrolyses a range of phosphodiesters. It is most active towards cyclic AMP, with a rate acceleration of ~10^9^ and a catalytic proficiency of >10^14^ M^−1^, comparable to larger enzymes shaped by billions of years of evolution.

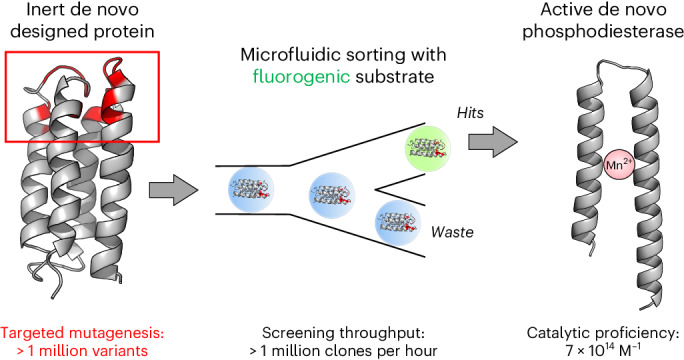

## Abstract (web summary)

Evolution separates complex modern enzymes from their hypothetical simpler early ancestors, which raises the question of how unevolved sequences can develop new functions. Here a library of non-natural protein sequences was subjected to ultrahigh-throughput screens in microfluidic droplets, leading to the isolation of a phosphodiesterase enzyme capable of hydrolysing the biological second messenger, cyclic AMP.

## Main

Living systems depend on chemical reactions that, if uncatalysed, occur too slowly to support life. Therefore, evolution has selected biological catalysts—enzymes—that speed up reactions to rates sufficient to sustain survival and growth. The enzymes found in present-day proteomes are typically well-ordered, finely tuned proteins, which provide rate enhancements far superior to enzyme catalysts created by design (with few exceptions so far^1^). Moreover, most enzymes in the current biosphere are relatively large, with bacterial and eukaryotic proteins having average lengths of 320 and 472 amino acids, respectively^2^.

It is relatively straightforward to envision how these large and highly active enzymes evolved by iterative selection for improvements of progenitor sequences that were already active and folded. However, it is far more challenging to understand how new functions were brought about in the first place. One may hypothesize that the first minimally active sequences arose de novo from inactive random polypeptides. Understanding how functional enzymes emerged from random sequences is particularly challenging in light of a large body of work showing that fully random sequences (1) rarely fold into well-ordered soluble structures^3,4^ and (2) rarely bind biologically relevant small molecules. For example, a seminal study by Keefe and Szostak^5^ showed that random libraries of 80-residue polypeptides include sequences that bind ATP at a frequency of approximately one in 10^11^. Catalysis is an even greater challenge than binding. The promiscuity of catalysts has been invoked to facilitate the emergence of new reactions after gene duplication and adaptive evolution of a side activity^6,7^. Metagenomic libraries have been found to contain catalysts for ‘unseen’, non-natural reactions with a xenobiotic substrate^8^ at a frequency of one in 10^5^. Eliciting function from non-catalytic sequences instead of re-functionalizing a (promiscuously) active enzyme is a much more difficult proposition, with very few examples on record and requiring the screening of libraries with more than 10^12^ members^9,10^. Computational design has been successful in principle^11^, but is far from routine, and creating high-efficiency catalysts immediately rivalling the efficiency of evolved enzymes has so far been difficult and is limited to a single case^1^, even though deep-learning-based algorithms are reinvigorating design approaches. Thus, in experimental and computational work, the step between inactive and active sequences seems a near-unsurmountable hurdle.

These considerations illustrate the tension between the abundant success of life on Earth and the extreme rarity of sequences capable of sustaining such life in the ‘vastness of sequence space’. Dayhoff had hypothesized^12,13^ that the first functional proteins emerged from short peptides (and their combinations by duplication, diversification and gene-fusion events during the course of evolution). The function of such primordial peptide building blocks then provides the historical link between prebiotic chemistry and the contemporary proteins of much larger sizes that eventually reached high efficiency and specificity. To probe the early emergence of function as a missing link to contemporary proteins, we set out to explore whether biologically relevant catalysts can be isolated from collections of unevolved de novo designed sequences. Because we anticipated that active catalysts would be rare and fully random sequences rarely fold into stable soluble structures^3,4^, several features were incorporated into the screen to enhance the likelihood of success. First, as a starting scaffold, we chose a collapsed globular structure that folds into a stable 4-helix bundle^14^. This scaffold, S-824, is a 102-residue protein (previously isolated from a semi-random library^15^) without natural function. Second, ultrahigh-throughput screening in microfluidic droplets (rate of ~0.8 kHz) was used to search libraries generated from S-824 for rare hits amidst a background of inactive sequences in a 1.7-million-membered library. Third, the screened substrates contained both a phosphodiester and a phosphotriester, thereby broadening the types of catalyst that might be discovered. Finally, a mixture of typical divalent metals (often found as cofactors in hydrolases) was added to the screen, thereby allowing the isolation of metalloenzymes derived from de novo design.

Our screen yielded a truncated and structurally dynamic 59-residue-long enzyme that accelerates phosphodiester hydrolysis in the presence of manganese ions by up to ~10^9^-fold over the uncatalysed background reaction. Promiscuous turnover was observed for phosphodiesters and phosphonates, including the unreactive substrate second messenger cyclic AMP (cAMP). The combination of an unevolved protein and a metal to process a nucleotide is prebiotically interesting—metals are thought to be highly important in prebiotic catalysis^16–18^, and phosphodiester bonds of nucleotides form the basis for information storage (DNA, RNA) and signalling (cAMP, cGMP). The rapid identification of a short protein that self-assembles into a dimeric structure with considerable activity provides support for the Dayhoff hypothesis^12,13^ and illustrates a scenario of rapid conversion of a peptide with no measurable activity into a proficient catalyst that is crucial for evolution.

## Results

### Microfluidic screening yields catalytically active proteins

**Fig. 1:**
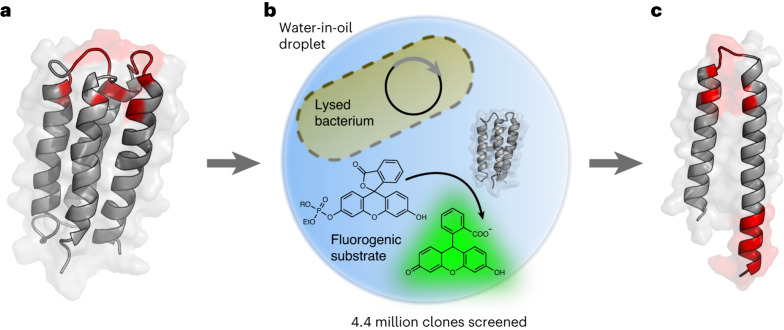
Droplet screening of a library of de novo designed 4-helix bundles enriches truncated sequences with phosphoesterase activity. **a**, De novo designed 4-helix bundle library. A library^20^ containing ~1.7 million variants based on the stably folded de novo designed 4-helix bundle protein S-824 was screened for phosphoesterase activity. The diversified residues of S-824 are shown in red (degenerate codons used: NDT/VRC/RRC). **b**, Ultrahigh-throughput microdroplet screening. The library was subjected to FADS on a microfluidic chip, and the 0.1–0.2% most fluorescent droplets (of a total of 4.4 million screened) were selected. **c**, Truncated peptide with phosphoesterase activity. The selection yielded catalytically active, truncated peptides consisting of a helix-turn-helix motif of ~60 amino acids (mutated sites are shown in red), illustrated here with a structural model of mini-cAMPase generated with AlphaFold2/ColabFold^32,34^. Panel **b** adapted with permission from ref. ^59^ under a Creative Commons licence CC BY 4.0.

The starting scaffold of S-824 is a stable 102-residue-long 4-helix bundle protein that had previously been isolated from a library randomized with binary patterning of polar and nonpolar amino acids^15,19^. S-824 is a rationally designed sequence of known three-dimensional structure (PDB 1P68)^14^ unrelated to any natural proteins. Such a 4-helix bundle scaffold tolerates enormous sequence diversity, with a hydrophobic core as the only minimal constraint^19^, minimizing the chances of sacrificing the folded structure and thus allowing us to interrogate the functional potential of diverse randomized sequences. S-824 was thus randomized in its apical loops and helix termini to create an active-site cavity surrounded by catalytic groups, producing a library of ~1.7 million variants (Fig. 1a)^20^.

The parental S-824 sequence showed no detectable phosphoesterase activity above background in our screening assay. To maximize the chances of capturing active enzymes, we incorporated diversity into all components of the screen. Not only were the sequences diverse, but we also screened for different catalytic activities simultaneously by using a bait mixture of fluorogenic phospho-di- and -triester substrates (Supplementary Fig. 1). Moreover, because many natural phosphoesterases utilize divalent metal cofactors, we increased the chance of finding a hit by supplementing the reaction with a mixture of MnCl_2_, ZnCl_2_ and CaCl_2_. We sampled all these variables combinatorially in an ultrahigh-throughput manner using microfluidic droplets, which allowed screening of the entire library in a single experiment. In this microfluidic assay, individual bacterial cells expressing a single library sequence were co-compartmentalized with the mixture of fluorogenic substrates and metals, and then lysed in the droplet (Fig. 1b and Supplementary Fig. 2)^21,22^. After incubation, the droplets were dielectrophoretically sorted at ultrahigh throughput by fluorescence-activated droplet sorting (FADS) on a microfluidic chip^23^. Sorted hits were subsequently identified by recovering and sequencing their plasmid DNA.

For the initial sort, we chose permissive conditions to maximize the number of screened clones and enhance the likelihood of finding hits. In total, 10.3 million droplets (corresponding to ~4.4 million clones) were screened in 4 h, and the 0.2–0.5% most fluorescent droplets were selected. The collected hits were clonally expanded and re-sorted under more stringent conditions to further enrich catalytically active clones. After this cumulative enrichment by droplet screening, ~250 clones were arbitrarily picked for secondary screening in microtitre plates. Average activity levels with the substrate mix increased with each round of sorting (Supplementary Fig. 3), indicating that screening cumulatively enriched sequences with phosphate hydrolase activity.

### Enrichment of truncations along with gain of function

**Fig. 2:**
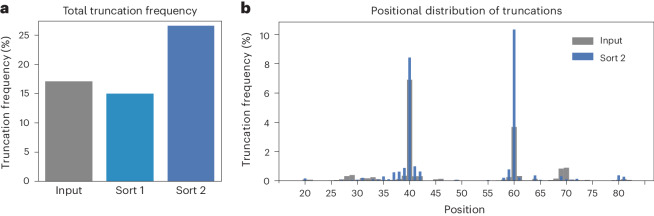
NGS analysis reveals enrichment of truncated proteins across the library. **a**, Relative frequency (percentage of truncated sequences among the total number of sequences) of truncated reads in the input library (grey; 17%), after sorting 1 (light blue; 15%) and after sorting 2 (dark blue; 27%) reveals 1.6-fold enrichment of premature stop codons after sort 2, albeit close to 0% would be expected. **b**, The frequency of truncations at every sequenced position in the input library (grey, broad bars) and after sorting 2 (blue, narrow bars) shows 1.2- and 2.8-fold enrichments of truncations at position 40 and 60, respectively, after sorting 2. Source data

**Extended Data Fig. 1:**
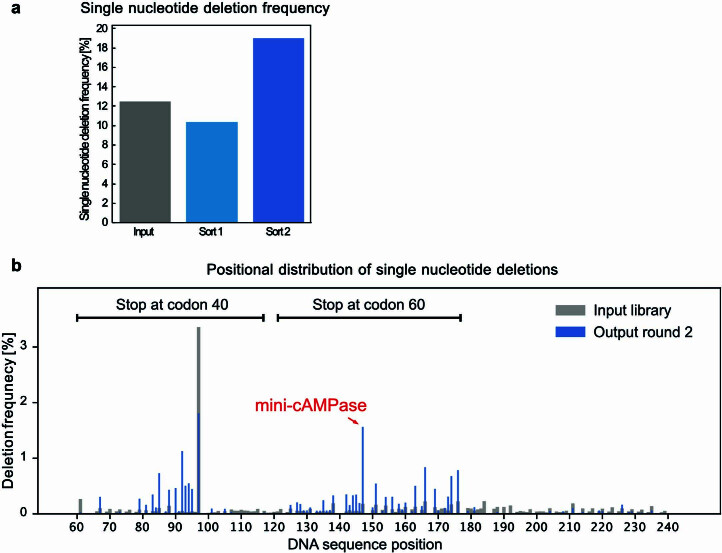
Enrichment of single-nucleotide deletions causing frameshifts after sorting 2. (**a**) Relative frequency (percentage of sequences including one single nucleotide deletion of total number of sequences) of single nucleotide deletions in input library (grey, 12%), after sorting 1 (light blue, 10%) and after sorting 2 (dark blue, 19%) reveals 1.6-fold enrichment of single nucleotide deletions after sorting 2. (**b**) Frequency of single nucleotide deletions at every sequenced position in input library (grey, broad bars) and after sorting 2 (blue, narrow bars). Deletions between position 55 and 117 cause a stop codon at codon 40 while deletions between position 121 and 177 cause a stop codon at codon 60. Deletions at position 147 (as found in the variant mini-cAMPase) are enriched 8.7-fold (0.18 vs 1.56%). Source data

Sequence analysis of these single hits from the secondary screen revealed that some of the most active sequences (12/14) had frameshift mutations that produced truncated protein sequences (Fig. 1c). Indeed, next-generation sequencing (NGS) analysis of the entire library before and after screening (~1.4 million unique variants) suggested that droplet screening had enriched single-nucleotide deletions along with the gain of function. These deletions led to frameshift mutations and consequently an enrichment in premature stop codons (from 17% to 27%; Fig. 2a and Extended Data Fig. 1). This observed enrichment stands in stark contrast to the fact that intra-gene stop codons are typically strongly selected against, as they usually lead to a loss of structure and function^24–26^. Thus, the paradox of an observed enrichment of truncated variants—as a general trend across the library—indicated that truncations might contribute to the selected phosphoesterase activity. Further analysis of their positional distribution revealed a high abundance of truncations at positions 40 and 60, with 18% of all sequences being truncated at one of these positions after the second sorting round. Truncations at both positions are enriched compared to the input library (1.2- and 2.8-fold; Fig. 2b). These truncated sequences lack the two C-terminal helices of the 4-helix bundle (Fig. 1c) and form the template for a shorter helix-loop-helix motif. Cysteine residues (which are absent in the S-824 ancestor) were also enriched, but their enrichment can be explained as a byproduct of frameshifts, co-introducing them into the coding sequence before the stop codons (Supplementary Fig. 4).

### A manganese-dependent de novo phosphodiesterase

**Fig. 3:**
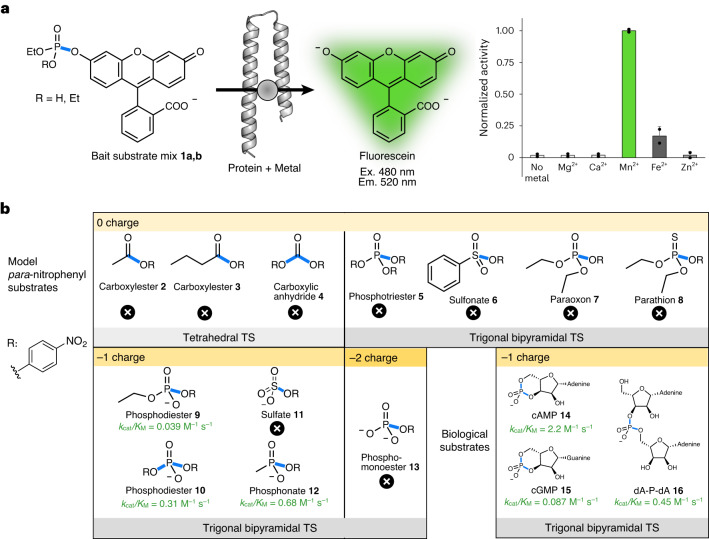
Metal requirement and substrate scope. **a**, Metal dependence of the enzymatic reaction for the mixture of fluorogenic bait substrates: 100 µM mini-cAMPase was incubated with ~200 µM substrate mixture and 200 µM Ca^2+^, Mg^2+^, Mn^2+^, Fe^2+^ or Zn^2+^. The histogram shows that mini-cAMPase requires Mn^2+^ for its catalytic activity. Data are presented as mean values of biological duplicates (*n* = 2), with error bars representing ±1 s.d. **b**, Activity was tested against substrates sampling different ground-state charges (from 0 to −2) and transition-state (TS) geometries (tetrahedral and trigonal–bipyramidal). The mini-cAMPase hydrolyses *p*-nitrophenyl phosphodiesters and phosphonates. The highest activity was observed for cAMP hydrolysis. The scissile bond is highlighted in blue. Cross symbols indicate no detectable activity. R indicates the *p*-nitrophenol leaving group. Source data

**Extended Data Fig. 2:**
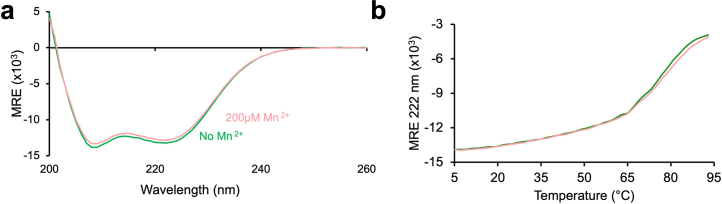
Metal binding effects on structure and thermal stability by Circular Dichroism (CD). (**a**) Full CD spectra of 40 µM mini-cAMPase in TBS without added Mn^2+^ (green) and with added 200 µM Mn^2+^ (pink). (**b**) Melting curves of the same samples as in (**a**), with and without manganese. Source data

We focused on analysing one clone (named mini-cAMPase hereon) with high catalytic activity, strong expression and good solubility, and disentangled the combinatorial elements of the screen (multiple substrates; multiple metal cofactors). Manganese was required for activity (Fig. 3a) while not altering the thermal stability (Extended Data Fig. 2), consistent with a catalytic rather than structural role. However, manganese binding could not be measured by isothermal titration calorimetry (ITC; Supplementary Fig. 5), which prevented determination of affinity and stoichiometry.

Next, we probed the substrate specificity by challenging the novel enzyme with a range of model substrates containing *p*-nitrophenyl leaving groups and encompassing a range of ground-state charges and transition-state geometries. As shown in Fig. 3b, these experiments revealed that the novel enzyme catalyses the hydrolysis of phosphodiester and phosphonate substrates carrying a single negative charge. Furthermore, activity was limited to substrates hydrolysed through a trigonal–bipyramidal transition state.

To assess activity beyond model compounds with nitrophenyl leaving groups, we extended the scope of the substrates to biologically important phosphodiesters. In contrast to the expectation that promiscuous activities identified in screens for thermodynamically undemanding substrates cannot easily be extended to more difficult reactions, we found that the novel enzyme catalyses the hydrolysis of less reactive biological phosphodiester substrates, cAMP and cGMP. It also shows some activity toward deoxyadenosine dinucleotide (dA-P-dA), which can be considered a simple model for DNAse activity (Supplementary Fig. 12). Because our novel protein is most active towards cAMP, we named it mini-cAMPase.

**Fig. 4:**
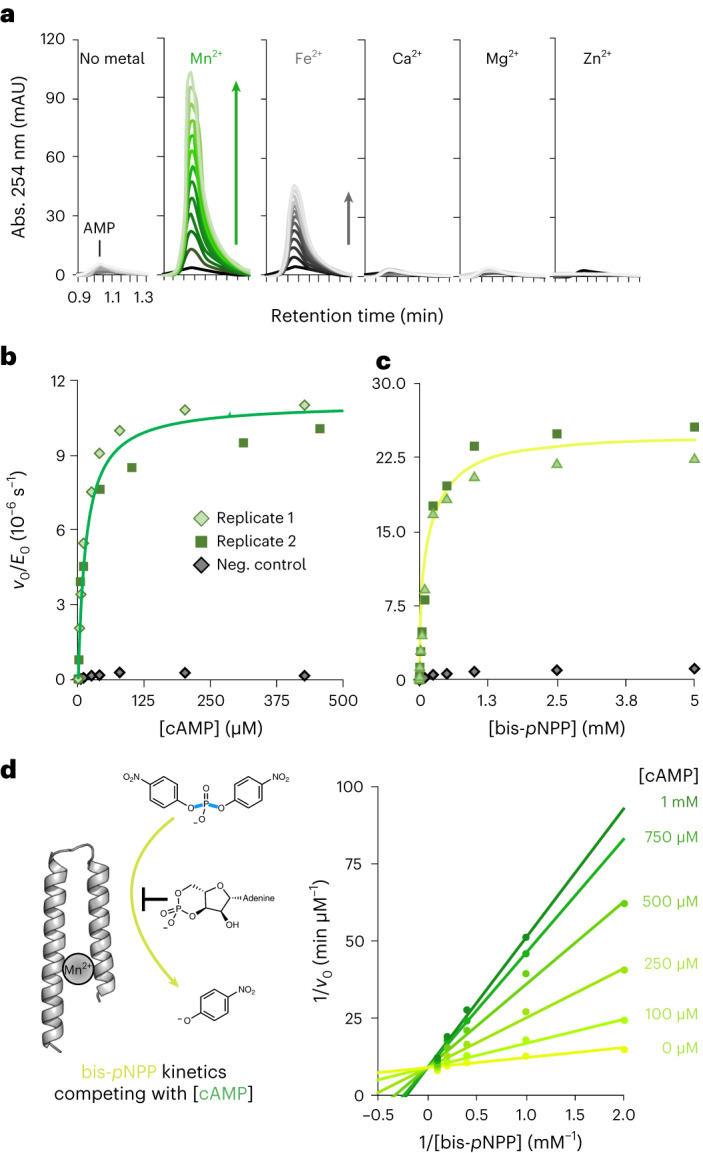
Kinetic characterization of enzymatic activity. **a**, Metal dependence of the mini-cAMPase activity. Spectral scans are shown that report on the appearance of AMP on reverse-phase (RP) HPLC (100 µM mini-cAMPase incubated with 100 µM respective divalent metal and 250 µM cAMP). Each successive trace is 1.5 h apart, with a total time of 18 h. **b**, Michaelis–Menten kinetics for cAMPase activity in biological duplicates with 50 µM protein and 200 µM MnCl_2_, measured at 25 °C in PBS (pH 7.4). The purification control (grey) shows the background activity for the same preparation steps on the protein without the His_6_-tag. Rates are the mean of three independent repeat datasets. Each colour shows a biological replicate. The purification control is shown in grey. **c**, Michaelis–Menten plot for the phosphodiesterase activity of 50 µM mini-cAMPase and 200 µM MnCl_2_ with the model substrate bis(*p*-nitrophenyl) phosphate in biological duplicates. Each colour shows a biological replicate. As before, the purification control is shown in grey. **d**, Competition between bis-*p*NPP and cAMP hydrolysis (left) shown in a Lineweaver–Burke plot (right). Lines connect kinetics for a single cAMP concentration, with higher cAMP concentrations shown in darkening hues of green. The shared *y* intercept indicates competitive inhibition with *K*_i_ = 70 ± 8 µM cAMP. Michaelis–Menten kinetics were assayed for bis(*p*-nitrophenyl) phosphate hydrolysis by 100 µM protein with 200 µM MnCl_2_ in increasing cAMP concentrations. Source data

**Extended Data Fig. 3:**
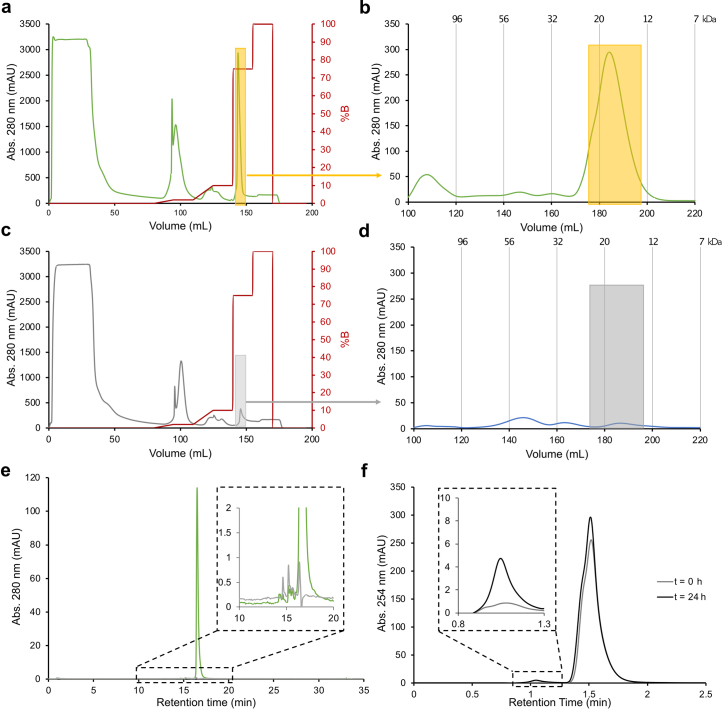
Controls for purification of mini-cAMPase. (**a**) Purification of (His)_6_-tagged mini-cAMPase on the nickel-NTA column, with the taken fractions boxed in yellow. These are then put on (**b**) the preparation-scale sizing column, where the fractions containing the protein are taken (again in yellow). To control for any endogenous proteins that come from this purification steps, in the same cellular background, the un-tagged protein was expressed alongside with the same fractions taken from the nickel-NTA column (**c**) and sizing column (**d**) here shown as grey boxes. (**e**) The samples with (green) and without (grey) the (His)_6_-tag are compared by HPLC, with the main peak being the protein mini-cAMPase. Inset is the magnified baseline. (**f**) The raw data for a single data point of the purification background control in Fig. 4b, c. The inset is the appearance of AMP. 250 µM cAMP was incubated with 200 µM Mn^2+^ in the background solution. This plot lacks concentration units but is diluted only slightly by the addition of cAMP and Mn^2+^. Note that, in contrast to the purified protein fraction, the control fraction was not diluted for this assay. Therefore, we estimate that the background activity represents an overestimate as any potential endogenous contaminants in the control fraction would be 2-fold to 5-fold more concentrated in the control fraction as compared to the protein fraction. These are the same conditions used for cAMPase activity characterization in Fig. 6. The very low observed activity in the control fraction indicates that the observed activity of mini-cAMPase cannot be attributed to an endogenous contaminant. Source data

**Extended Data Fig. 4:**
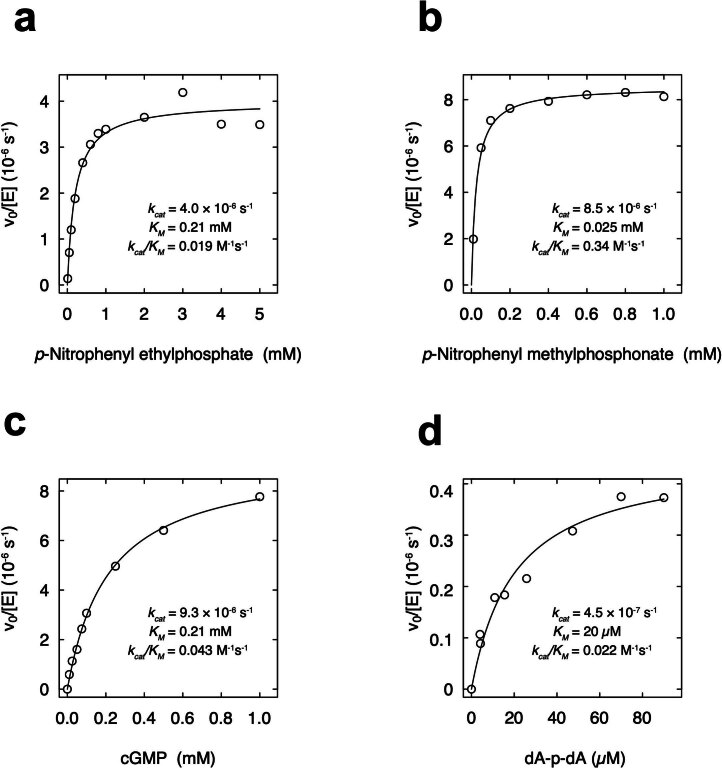
Michaelis-Menten plots for kinetics of mini-cAMPase. Steady-state kinetics were measured with **(a)** *p*-nitrophenyl ethylphosphate **(b)** *p*-nitrophenyl methylphosphonate, **(c)** cGMP, and **(d)** dA-P-dA. Protein was lyophilized after HPLC, and resuspended in 50 mM HEPES-NaOH, 150 mM NaCl, 5 mM DTT, pH 8.0 at 25 °C. Curves with cAMP and bis-*p*NPP are shown in Fig. 4b, c. Source data

The observation of catalytic activity for a de novo protein expressed in *Escherichia coli* inevitably raises concerns about the possibility of contaminating activity from endogenous proteins^27,28^. We ruled out this possibility by performing several control experiments, showing that the observed activity cannot be attributed to endogenous *E. coli* cAMPase (CpdA)^29,30^ or other contaminating proteins. First, mini-cAMPase requires a different metal cofactor and has a much lower Michaelis constant than endogenous *E. coli* cAMPase. Second, the enzymatic activity co-purifies with the mini-cAMPase fraction, independent of the purification method. Third, sequence changes in mini-cAMPase correlate with changes in enzymatic activity. These control experiments thus provide evidence that mini-cAMPase is a de novo phosphodiesterase (for details, see note 1.5 in the Supplementary Information; Fig. 4a–c and Extended Data Figs. 3 and 4).

### Kinetic characterization and evidence for a common active site

**Extended Data Fig. 5:**
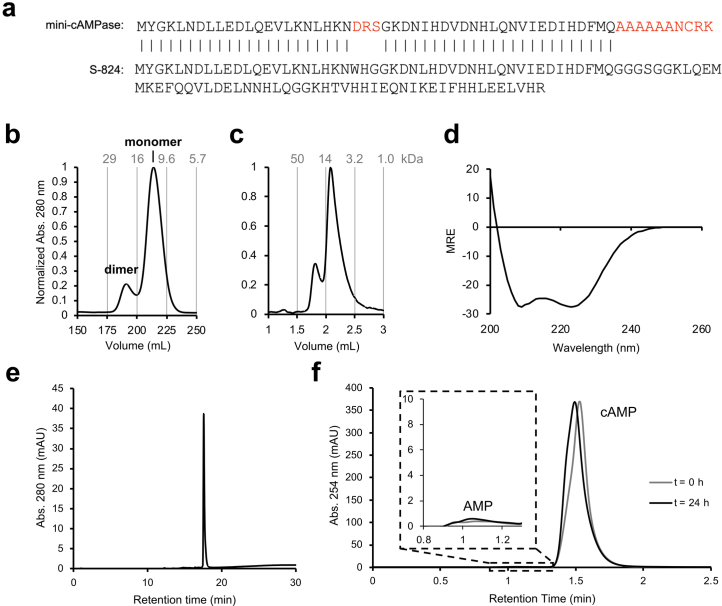
S-824 purification and lack of activity. (**a**) Sequence comparison of characterized hit protein and S-824 with mismatches highlighted in red. (**b**) Preparation-scale and (**c**) analytical-scale size-exclusion chromatography shows that S-824 exists predominantly as a monomer, but a dimer is partially present. (**d**) Circular Dichroism spectra of S-824 in TBS shows that it is helical. (**e**) Reverse-phase HPLC of S-824 shows that it is of high purity (>99%). (**f**) The raw data for a single data point in Extended Data Fig. 7b, sampled before and after 24 h for 50 µM S-824 incubated with 200 µM Mn^2+^ and 250 µM cAMP (the same conditions used for cAMPase activity characterization in Extended Data Fig. 7b), showing that S-824 lacks cAMPase activity. Source data

Kinetic characterization of mini-cAMPase with both model and biological substrates revealed the largest catalytic efficiency (*k*_cat_/*K*_M_) of 2.2 M^−1^ s^−1^ for cAMP, which corresponds to a rate acceleration of ~7 × 10^9^ over the uncatalysed background reaction (in the absence of manganese) and a catalytic proficiency of ~7 × 10^14^ M^−1^ (Table 1 and Fig. 4b,c). Because of this high activity and biological relevance, cAMP hydrolysis became the focus of our further characterization. As with the fluorogenic substrate mixture, we found that mini-cAMPase best hydrolyses cAMP in the presence of manganese and, to a lesser extent, iron (Fig. 4a). The turnover is slow, but kinetic measurements yield a well-defined Michaelis–Menten profile for both the diesters cAMP and bis-*p-*nitrophenyl phosphate (bis-*p*NPP) (allowing calculation of the maximal velocity *V*_max_ and suggesting active-site saturation). Notably, the parental sequence S-824 lacks detectable cAMPase activity (Extended Data Figs. 5f and 7b) but shows low activity towards bis-*p*NPP (*k*_cat_/*K*_M_ ≈ 4 × 10^−3^ M^−1^ s^−1^, so ~80-fold lower than mini-cAMPase; Extended Data Table 1 and Extended Data Fig. 7c).

**Table 1:** Michaelis–Menten constants for mini-CAMPase with phosphonate and phosphodiester substrates

Substrate	*k*_cat_ (10^−6^ s^−1^)^d^	*K*_M_ (µM)^d^	*k*_cat_/*K*_M_ (M^−1^ s^−1^)	*k*_cat_/*k*_uncat_	(*k*_cat_/*K*_M_)/*k*_uncat_ (M^−1^)
*p*-nitrophenyl-methylphosphonate^a^	17 ± 0.2	25 ± 3	0.68	1 × 10^6^	4 × 10^10^
*p*-nitrophenyl-ethylphosphate^b^	8.0 ± 0.2	210 ± 31	0.039	1 × 10^10^	6 × 10^13^
bis(*p*-nitrophenyl-)phosphate ^b^	50 ± 4	160 ± 40	0.31	7 × 10^10^	5 × 10^14^
cAMP^c^	22 ± 4	10 ± 3	2.2	7 × 10^9^	7 × 10^14^
cGMP^c^	19 ± 0.4	210 ± 10	0.087	6 × 10^9^	3 × 10^13^
dA-P-dA^b^	0.91 ± 0.1	20 ± 4	0.045	1 × 10^9^	6 × 10^13^

^a^Based on *k*_uncat_ = 1.7 × 10^−11^ s^−1^ for *p*-nitrophenyl phenylphosphonate at pH 7.5 and 30 °C (ref. ^80^).

^b^Based on *k*_uncat_ = 7 × 10^−16^ s^−1^ for P–O cleavage in dineopentyl phosphate at pH 7 and 25 °C (ref. ^81^).

^c^Based on *k*_uncat_ = 3 × 10^−15^ s^−1^ for P–O cleavage in ethylene phosphate (a cyclic phosphodiester) at pH 7 and 25 °C (ref. ^82^). For further details, see note 1.6 in the Supplementary Information.

^d^The indicated error is the error of the nonlinear fit to the Michaelis–Menten equation.

Although both the model and biological substrates (Fig. 3b) are phosphodiesters, they have very different structures. Therefore, we were curious about whether mini-cAMPase hydrolysed these substrates using the same active site. To address this question, we probed whether hydrolysis of the model chromogenic substrate bis-*p*NPP could be inhibited by cAMP. As shown in Fig. 4d (and elaborated in Supplementary Fig. 6), hydrolysis of bis-*p*NPP in the presence of increasing cAMP concentrations showed competitive inhibition with a *K*_i_ of 70 ± 8 µM for cAMP. These results indicate that, despite their structural and electronic differences, both substrates rely on the same active site.

### Mutational and structural analysis of mini-cAMPase

**Extended Data Fig. 6:**
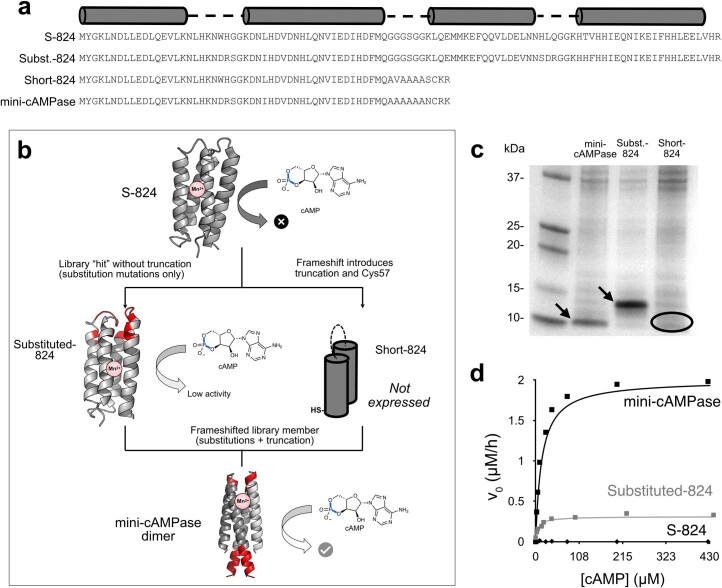
Substitutions and truncations contribute to phosphodiesterase activity. (**a**) Protein sequences showing the changes that bridge S-824 and mini-cAMPase. (**b**) Scheme of the relationship between the sequences and the effect of the truncation on function. (**c**) SDS-PAGE of whole cell extracts shows that Short-824 is poorly expressed (single measurement). (**d**) Michaelis-Menten kinetics comparing S-824, mini-cAMPase, and Substituted-824, showing that Substituted-824 is ≈ 6-fold less active (in *k*_*cat*_/*K*_*M*_) than the truncated mini-cAMPase protein. Source data

Mini-cAMPase carries both a truncation and substitutions when compared to S-824. To address the relative contributions of both factors to the observed catalytic activity, we tested two constructs (Extended Data Fig. 6). First, probing the importance of the truncation, we maintained the substitutions observed in mini-cAMPase but removed the frameshift mutation introducing the truncation. The resulting construct ‘Substituted-824’ preserves the full length of S-824, but contains mini-cAMPase’s mutations ahead of the frameshift mutation (Extended Data Fig. 6a). Substituted-824 expressed well and, in contrast to the parental sequence S-824, showed activity towards both phosphodiester substrates, but with a substantially reduced *k*_cat_ (~7-fold towards cAMP and ~17-fold towards bis-*p*NPP as compared to mini-cAMPase; Extended Data Fig. 6d). Second, to probe the role of the substitutions, we introduced the truncation, but not the substitutions, into the parental protein, producing ‘Short-824’ (Extended Data Fig. 6a). Short-824 expressed very poorly (Extended Data Fig. 6c), suggesting that the library substitutions contribute to stabilization of the truncated protein. These tests indicate that the library-designed substitutions and the unexpected truncation both contribute to identifying mini-cAMPase as a hit (Extended Data Fig. 6b).

**Extended Data Fig. 7:**
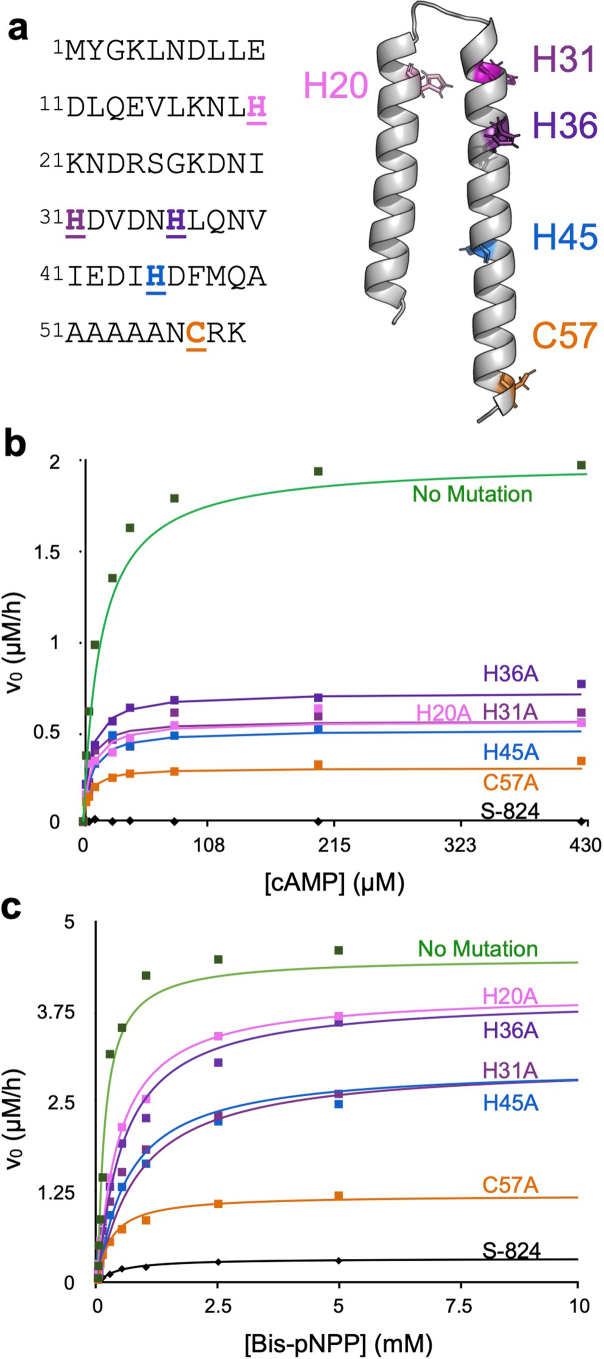
Impact of alanine mutations on the activity of mini-cAMPase. (**a**) Protein sequence and predicted structure with hydrophobic residues in yellow. Sites of mutated residues are emphasized and colour-coded. (**b**) cAMPase kinetics of alanine point mutants targeting the potential metal binding residues, alongside the inactive ancestor S-824 (black diamonds along baseline, with example raw data in Extended Data Fig. 5). (**c**) Bis-*p*NPP kinetics of alanine point mutants, alongside the inactive ancestor S-824. Note that panels (b) and (c) are plotted on different scales. Source data

To identify the residues directly involved in catalysis or binding of the Mn^2+^ cofactor, we mutated several residues individually to alanine and measured the Michaelis–Menten kinetics of the mutants (Extended Data Table 1). We targeted residues that might be involved in manganese binding, including histidine and cysteine, as well as the arginine consistently introduced by the truncations (Extended Data Fig. 7a). All introduced mutations reduced *k*_cat_, with C57A having the largest impact, lowering *k*_cat_ by ~6.5-fold towards cAMP and ~3.7-fold towards bis-*p*NPP (Extended Data Fig. 7b,c). This indicates that C57 is involved in, but not essential to, catalysis.

**Fig. 5:**
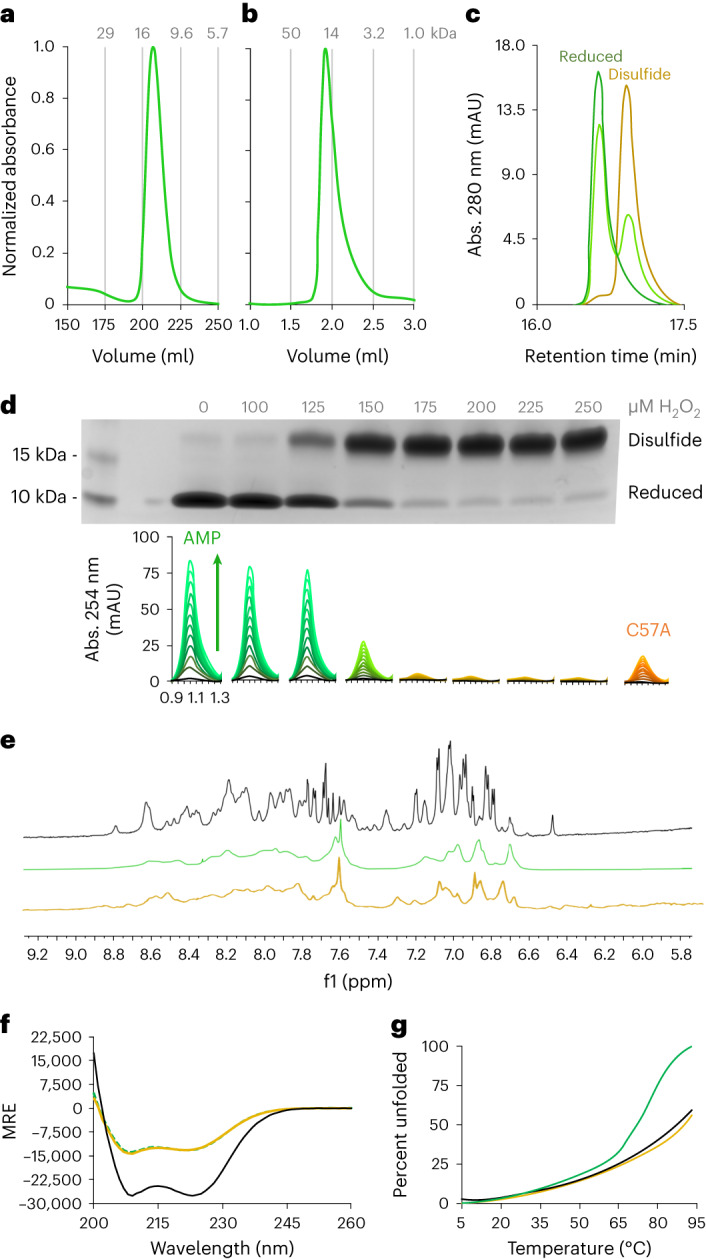
Mini-cAMPase is a dynamic α-helical dimer. Across all panels, yellow represents the disulfide-bonded dimer, green is the reduced protein, and black is the parental protein (S-824). **a**,**b**, Preparation-scale (**a**) and analytical-scale (**b**) SEC shows that reduced mini-cAMPase elutes at double its monomeric molecular mass (that is, it is a noncovalent dimer). **c**, RP-HPLC traces show reduced (green), partly oxidized (yellow–green, 125 µM H_2_O_2_) and fully oxidized (yellow, 250 µM H_2_O_2_) protein. **d**, Activity under a gradient of increasingly oxidizing conditions to promote disulfide formation. Non-reducing SDS–PAGE reveals the relative abundance of disulfides (single measurement). Below each gel lane we show the HPLC traces indicating the corresponding activity of 50 µM mini-cAMPase incubated with 200 µM Mn^2+^ and 250 µM cAMP. The HPLC traces monitor the appearance of AMP (assayed by RP-HPLC) measured every 2 h for 24 h. The mutant C57A is included for comparison. **e**, ^1^H NMR showing the amide region for S-824 (black) and mini-cAMPase (reduced, green; oxidized, yellow). The broad and poorly resolved peaks indicate that the protein does not form a well-ordered structure and remains dynamic, even after oxidation. **f**, CD spectra showing that mini-cAMPase (green, yellow) is helical, but less so than the parent 4-helix bundle (black). There is no discernible shift in secondary structure caused by oxidation of C57. MRE, mean residue ellipticity. **g**, Melting curves measuring CD at 222 nm show that oxidized mini-cAMPase (yellow, *T*_M_ > 90 °C) has a melting curve similar to S-824 (black, *T*_M_ > 90 °C), with both proteins more thermostable than the reduced mini-cAMPase (green, *T*_M_ ≈ 72 °C). Source data

Because only two of the original four helices are present, we surmised that mini-cAMPase might form a helical hairpin that dimerizes into a 4-helix bundle. Consistent with this expectation, size-exclusion chromatography (SEC) showed that mini-cAMPase elutes as a dimer (Fig. 5a,b). This dimer was formed under reducing conditions and confirmed by liquid chromatography mass spectroscopy (LC/MS) to be fully reduced at its only cysteine residue, C57 (Supplementary Fig. 11). Further experiments under oxidizing conditions showed that disulfide bridge formation also leads to dimerization but abolishes activity completely (Fig. 5c,d and Supplementary Fig. 11). Loss of activity implies that the disulfide-linked dimer structure is incompatible with efficient catalysis, although concomitant oxidation of the Mn^2+^ cofactor cannot be ruled out as a cause for inactivation. The pH dependence of enzymatic second-order rates (Supplementary Fig. 13) shows an apparent p*K*_a_ of 7.8, similar to the measured p*K*_a_ of a bona fide phosphodiesterase that also contains Mn^2+^ (ref. ^31^).

^1^H NMR and circular dichroism (CD) spectroscopy further indicate that mini-cAMPase is structurally less defined than its parental sequence S-824 (Fig. 5e–g), at least in the absence of manganese. This could indicate that it dynamically samples an ensemble of states, potentially including conformations that enable activity. Alternatively, in an induced fit model, metal-binding could potentially induce a more defined, functional structure in mini-cAMPase that is not sampled by the catalytically inactive ancestor S-824.

**Extended Data Fig. 8:**
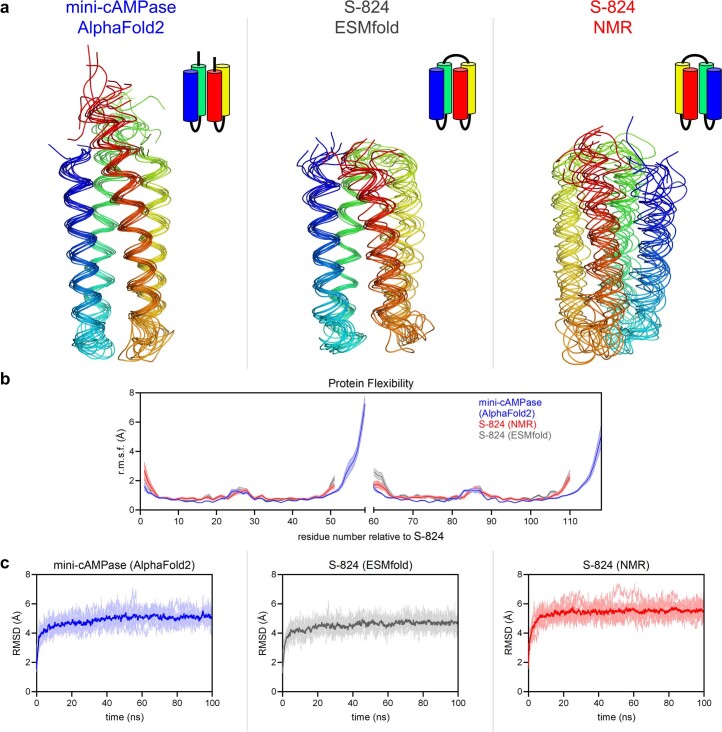
Molecular dynamics simulations with mini-cAMPase. **(a**) The dynamics of S-824 in either of the two observed topologies, as well as mini-cAMPase were analyzed by 100 ns molecular dynamics simulations. **(b**) Molecular dynamics simulations indicate that the mini-cAMPase helical-bundle core becomes slightly more rigid. In contrast, the C-terminal tails is highly dynamic, which could potentially explain the experimental observations from ^1^H NMR and CD spectroscopy. **(c)** RMSD plots indicate that the simulations are stable and converged.

**Extended Data Fig. 9:**
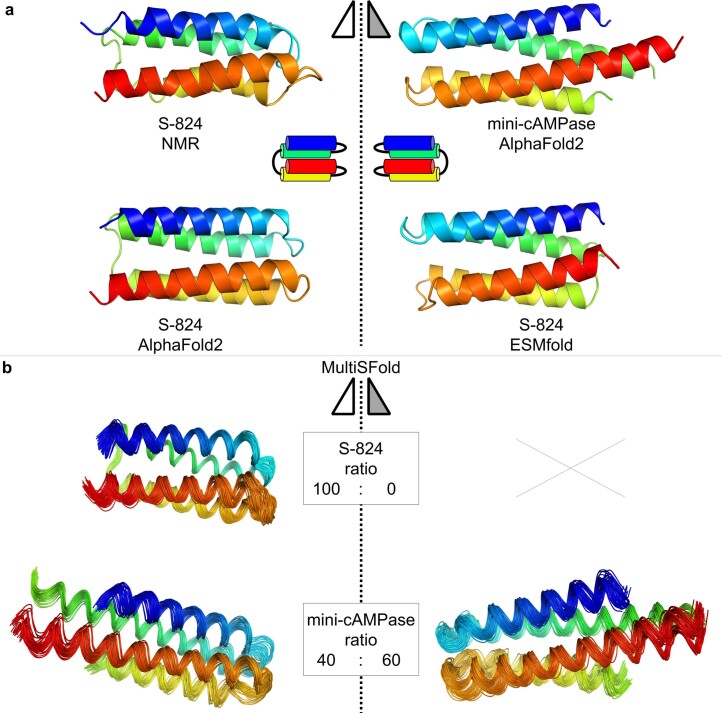
Structure prediction of S-824 and the mini-cAMPase dimer. **(a)** AlphaFold2 reproduced the NMR model of S-824. In contrast, the mini-cAMPase dimer adopts a different topological isomer in which the overall topology is mirrored. EMSfold^35^ predicted that S-824 populates that other topoisomer. **(b)** MultiSFold was used to dissect the topological dynamism further. For S-824, the topology observed in NMR was predicted exclusively. For mini-cAMPase, both topologies were observed in a ratio of 40:60. In addition to the more flexible C-terminus, the variability in topology could be another explanation for the experimental observation from ^1^H NMR and CD spectroscopy.

We performed molecular dynamics (MD) simulations to probe our hypothesis that changes in dynamics give rise to the functional changes observed in mini-cAMPase. On a timescale of 100 ns, no substantial difference in the per-residue fluctuations between the S-824 ancestor and the mini-cAMPase dimer were detected, apart from the mini-cAMPase dimer having a short, disordered C-terminal tail (Extended Data Fig. 8). Thus, the dynamic effects observed with ^1^H NMR and CD spectroscopy are likely to occur on a considerably slower timescale than the MD simulations. Interestingly, AlphaFold2^32–34^ reproduced the NMR model of S-824, but indicated that the mini-cAMPase dimer adopts a different topological isomer in which the overall topology is mirrored (Extended Data Fig. 9). Curiously, EMSfold^35^ predicted that S-824 populates the other topoisomer, leaving two candidate structures to be considered. To dissect possible topological dynamism, we turned to MultiSFold^36^, a computational framework aimed at predicting conformational isomers. MultiSFold^36^ predicted a single isomer resembling the NMR structure for S-824 but a 40:60 ratio of the mini-cAMPase topoisomers. This conformational diversity is consistent with the observations from ^1^H NMR and CD spectroscopy and could indicate that mini-cAMPase exists in an equilibrium of distinct dimeric conformations that adopt different topologies and exchange slowly (on or greater than a microsecond timescale).

## Discussion

Mini-cAMPase has several notable features. (1) It was isolated from a library of variants that is relatively small (~1.7 × 10^6^) compared to the vast sequence space available to natural evolution (20^102^ ≈ 10^132^ for a protein of the same length as S-824 or 10^78^ for a 59-residue mini-cAMPase). (2) Although the parental S-824 shows no phosphoesterase activity in our lysate assays, the selected mini-cAMPase recruits a metal cofactor to catalyse phosphodiester hydrolysis with high catalytic proficiency. (3) Although the inactive parental sequence was 102 residues long, screening for activity led to a truncation protein of 59 residues, approximately half the length of the parent. (4) Although the S-824 parent is a well-ordered monomeric 4-helix bundle, the selected mini-cAMPase enzyme forms a 2 × 2 helix dimer with increased flexibility after loss of a covalent constraint imposed by the original single-chain protein.

Given that mini-cAMPase has no evolutionary history, its catalytic efficiency for cAMP hydrolysis (*k*_cat_/*K*_M_ ≈ 2.2 M^−1^ s^−1^; Table 1) is remarkable, being only ~1,000-fold lower than that of, for example, the native cAMPase (CpdA) from *E. coli* (Supplementary Table 3). Put in the broader context of the average natural enzyme that is acting on its preferred substrate (*k*_cat_/*K*_M_ ≈ 10^5^ M^−1^ s^−1^)^37^, mini-cAMPase is orders of magnitude less active. However, its *k*_cat_/*K*_M_ is only ~14-fold lower than the median value of 31 M^−1^ s^−1^ reported for natural enzymes catalysing a promiscuous reaction^38^, and a ‘head start’ activity^7^ as the basis of further evolution is conceivable, enabling rounds of directed evolution. Although the first-order rate constant *k*_cat_ of mini-cAMPase lags behind that of naturally occurring phosphodiesterases specific for cAMP (2.2 × 10^−5^ s^−1^ versus 10^−1^ to 10^3^ s^−1^; Supplementary Table 3), mini-cAMPase displays a substantial rate enhancement (*k*_cat_/*k*_uncat_) of 7 × 10^9^ and a catalytic proficiency (*(k*_cat_/*K*_M_)/*k*_uncat_) in the range of 7 × 10^14^ M^−1^ over the uncatalysed background reaction (Table 1). These parameters are comparable to those reached by some enzymes with their native substrates, although higher (and lower) values are on record^39^. Our work shows how catalysis of a difficult biologically relevant reaction with accelerations (*k*_cat_/*k*_uncat_) approaching those of large natural enzymes selected by billions of years of evolution can be brought about in an efficient screen of a million-membered library.

While representing only a small fraction of all possible diversity, the successful outcome of the screen emphasizes that Dayhoff was not wrong about the odds for finding catalysts, even in such scenarios of partial sampling of sequence space. mini-cAMPase is a rare example of rapid functionalization of a short, inactive peptide, validating a broadened version of the Dayhoff hypothesis^13^ and dismissing the sceptical (or creationist) view that the sequence space for peptides with no biological ancestry does not hold sufficient solutions for catalytic challenges. In particular, Dayhoff’s notion that assemblies of primordial peptides (for example, generated by duplication) can aquire functions is reflected in our findings, even though in our case a dimer is generated by truncation of a longer peptide, in contrast to Dayhoff’s original work focusing on duplication of a short peptide^12^. In this reverse mode of evolution (that is, from larger peptides to smaller ones), the effect of disassembly may be analogous to the effect of insertions and deletions (InDels) as motors of evolution by enabling disruptive but innovative changes that help the acquisition of function^40–42^. Specifically, the evolution of a well-packed, designed protein may need departure routes to structural, topological and conformational alternatives that hold catalytic solutions (as evidenced in our MD simulations; Extended Data Figs. 8 and 9).

Our work advances previous research studying the catalytic activity of semi-random 4-helix bundles as de novo designed proteins. For example, mini-cAMPase acts on a more thermodynamically challenging substrate than our previously reported 4-helix bundle ATPase, and does so with a higher *k*_cat_ (ref. ^43^). Its small size is also noteworthy, being roughly half of our previously reported dimeric 4-helix bundle that hydrolyses enterobactin^44,45^. The lack of a designed metal-binding centre also stands in contrast to previous work looking at designed metalloenzymes^46,47^; unlike many designed metalloenzymes, mini-cAMPase makes use of Mn^2+^ largely by serendipity. The catalytic proficiency of mini-cAMPase (~10^14^ M^−1^; Table 1) goes beyond previous models: for example, a partly designed and evolved 4-helix bundle metalloprotein with a catalytic proficiency of 9.3 × 10^10^ M^−1^ for carboxyesters^48^ further designed and evolved into a Diels–alderase^49^ with a catalytic proficiency of 2.9 × 10^11^ M^−1^. Similarly, a previously reported^50^ de novo helix-turn-helix peptide that dimerizes into a 4-helix bundle hydrolyses the phosphodiester *p*-nitrophenyl phosphate (in the absence of metal) with a second-order rate constant of 1.58 × 10^−4^ M^−1^ s^−1^, resulting in a catalytic proficiency of ~2 × 10^11^ M^−1^, which is three orders of magnitude lower than for the mini-cAMPase. mini-cAMPase also surpasses the rate enhancement of a zinc-based biomimetic phosphodiesterase model by five orders of magnitude^51^. These distinguishing features make mini-cAMPase quite unlike previously characterized minimalist enzyme models.

Intriguingly, the recruitment of a divalent metal cofactor for phosphodiester hydrolysis by mini-cAMPase recapitulates the evolution of naturally occurring phosphodiesterases. Extant, naturally occurring cAMP-hydrolysing enzymes have emerged independently in three different folds, converging towards mechanisms that involve a divalent metal ion cofactor^52^. Most natural phosphodiesterases use Zn^2+^, and the *E. coli* cAMPase (CpdA) requires Fe^2+^ or Mg^2+^ (ref. ^29^). In contrast, mini-cAMPase requires Mn^2+^ for activity, suggesting an unprecedented variation to the theme of divalent metal-ion catalysis.

The strategy of starting a combinatorial screen by randomizing a stable fold followed by ultrahigh-throughput screening with multiple cofactors and substrates has resulted in an enzyme with catalytic proficiency surpassing that of most de novo designed and evolved proteins by several orders of magnitude. The isolation of mini-cAMPase from a relatively small library (compared to the theoretical size of the sequence space) was facilitated by two factors. First, the use of microfluidic droplet sorting allowed screening for multiple-turnover catalysis at very high throughput (4.4 million clones screened in just 4 h), ensuring that most clones of our million-membered library were sampled at least once (~2.6-fold oversampling). Microfluidic droplet screening has been used previously to evolve or enrich (mostly hydrolytic) enzymes with known activity from large libraries by fluorescence^8,53–60^ or other detection modes^61,62^, but has never been applied to a library of de novo designed proteins with unknown function. Our findings suggest that targeted randomization of previously inactive scaffolds, when analysed by ultrahigh-throughput technologies, provides unexpected catalytic solutions (for example, truncation) that complement the lessons about the acquisition of function in contemporary enzymes. Previous examples of generating function from inactive scaffolds also relied on ultrahigh-throughput screening directly for catalysis, like mRNA display, albeit with much larger >10^12^-membered libraries^9,63^. Second, targeted randomization of a de novo sequence designed by binary patterning to fold into a stable 4-helix bundle enhanced the number of sequences in the library displaying a stable fold^20^.

Unexpectedly, selection for functional catalysts enriched library members that ‘escape’ the pre-defined stable 4-helix bundle fold by truncation to specific lengths, forming a helix-turn-helix motif. Although such major truncations are usually expected to be detrimental to protein function, NGS confirmed this as a general trend across the entire library rather than a mutational accident (Fig. 2). As characterized in detail for the case of mini-cAMPase (Extended Data Figs. 8 and 9), the remaining 59 residues maintain the binary patterning of the original design. Observing a dimer formed of α-helices (Fig. 5a,b) suggests a return to a 4-helix bundle fold, albeit with additional degrees of freedom that may employ dynamic effects in binding and catalysis (Fig. 5e–g). It has been pointed out that the appearance of symmetrical structures is favoured as a low-complexity (high-symmetry) phenotype^64^ due to the fact that symmetrical structures are stochastically more likely to appear and are therefore predicted to prevail due to an arrival-of-the-frequent mechanism^65^. In analogy to our findings, ‘creative destruction’ has been proposed as a mechanism for the emergence of new protein folds from existing, simpler structures^66^.

The selection of a truncated library member invites speculation that the 4-helix bundle fold of the library ancestor S-824 is very stable but structurally limited, as its well-folded state was designed to occupy a distinct energetic minimum in the protein folding funnel. Compared to its parent S-824, the structure of mini-cAMPase is highly dynamic (Fig. 5e–g), possibly fluctuating between multiple conformational states or dimeric arrangements. This feature might represent a departure from S-824’s ‘frozen’ conformation in a deep thermodynamic well to a less well-defined folding landscape that can select alternative conformations conducive to catalytic function^67–69^. Sampling new conformational states or different topologies may also provide functional innovation that can be further enriched in subsequent steps of evolution^70,71^. The disorder seen in mini-cAMPase can thus be likened to a catalytically proficient molten globule enzyme^72^ or the melting of a zinc finger that conferred ligase activity to a non-catalytic scaffold^10^. Alternatively the dynamic behaviour may simply be a sign of damage incurred by mutation^70,71^, where structural disorder is responsible for ineffecient catalysis, even though the disruption of structure may be necessary to bring about a new function in an existing scaffold^73,74^.

Evolved natural enzymes typically fold into large well-ordered structures with pre-organized active sites. Presumably, these were selected to favour highly active and substrate-specific specialists that can be allosterically regulated. In the early stages of evolution, however, it may have been advantageous to express dynamic sequences that sampled an ensemble of states. Although these molten structures probably had low activities, they may have been able to catalyse several chemical reactions, acting on a range of substrates. Such multifunctional generalists would have enhanced the ‘catalytic versatility of an ancestral cell that functioned with limited enzyme resources’^6,75^. In addition, small, dynamic proteins may have advantages as starting points for further evolution and adaptation. Although large modern enzymes are restricted by an epistatic burden that causes mutations to interfere with structural and functional innovation^76–79^, small dynamic proteins could escape the limits imposed by cooperative effects and become functionally more versatile and more evolvable by reducing the cost of innovation. These patterns may reflect the role of smaller, structurally versatile peptides in Dayhoff’s model^12,13^. Here, functional proficiency was found by seemingly going back to the origins of life, paradoxically reaching improvements in primitive rather than sophisticated scaffolds that, as low-probability events, nevertheless become accessible via ultrahigh-throughput screening.

## Methods

### Library preparation

The library encoding the partly randomized de novo designed gene *S-824* was cloned from the original pET11a vector^20^ into the high-copy-number vector pASK-IBA5+ (IBA Life Sciences) using the restriction sites XbaI and BamHI. This allowed highly efficient DNA recovery by transformation after droplet sorting as well as strain-independent tetracycline-inducible expression^83^.

### Microfluidic library screening

Microfluidic library screening was carried out as previously described in detail in ref. ^59^. In brief, the library was electroporated into *E. coli* cells (E. cloni 10G Elite; Lucigen), yielding ~10^7^ colonies after overnight incubation on agar plates, as determined by serial dilution. After induction and incubation for protein expression, the cells were washed in HEPES buffer (50 mM HEPES-NaOH, 150 mM NaCl, pH 8.0) and encapsulated together with the substrate mixture (~100 µM), metal mixture (MnCl_2_, ZnCl_2_ and CaCl_2_ at 10 µM, respectively) and lysis agent in monodisperse water-in-oil droplets on a flow-focusing chip (Supplementary Fig. 2a), generating droplets with a volume of 3 pl at rates of 0.5–3 kHz. The droplets were collected in a closed storage chamber, fabricated from an eppendorf tube as previously described^84^. The microfluidic devices were fabricated by soft lithography as previously described^59^. After incubation at room temperature, the droplets were reinjected from the collection tubing into the sorting device (Supplementary Fig. 2b). In contrast to the previously described sorting device^59^, this chip featured an additional flow of ‘bias oil’ from the side that forced the droplets further away from the hit channel at a flow rate of 30 µl h^−1^, reducing the number of false-positive droplets accidentally flowing into the hit channel. Droplets were sorted according to their fluorescence at a rate of ~0.8 kHz and collected into a tube pre-filled with water. After sorting, the hit droplets were de-emulsified by the addition of 1*H*,1*H*,2*H*,2*H*-perfluorooctanol (Alfa Aesar), the solution was purified and concentrated by column purification (DNA Clean & Concentrator-5 kit, Zymo Research), and the plasmid DNA was recovered by electroporation into highly electrocompetent *E. coli* cells, as previously described in detail^59^. In the initial sorting, permissive conditions were chosen: the cells were encapsulated at an average droplet occupancy (*λ*) of 0.43 and the droplets were incubated for three days. Out of 10.3 million screened droplets, 53,000 droplets were sorted (approximately the top 0.5%). In the second sort, screenings were conducted under more stringent conditions, with cell encapsulation at *λ* = 0.1, and the droplets were incubated for seven days. Of 4.4 million screened droplets, 7,500 droplets were sorted (approximately the top 0.2%).

### Microtitre plate screening

To quantify the lysate activity of the library variants, individual colonies were picked and grown in 96-deep-well plates in 500 μl of Lysogeny broth (LB) medium (containing 100 μl ml^−1^ carbenicillin) at 37 °C/1,050 r.p.m. for 14 h. Subsequently, 20 μl of overnight cultures were used to inoculate 880 μl of Terrific broth (TB) medium (containing 100 μl ml^−1^ carbenicillin and 2 mM MgCl_2_) for expression cultures in 96-deep-well plates, which were grown at 37 °C/1,050 r.p.m. for 2–3 h until reaching an optical density at 600 nm of ~0.5. Expression was then induced with anhydrotetracycline (final concentration 200 ng ml^−1^; IBA Life Sciences) and carried out for 16 h at 20 °C and shaking at 1,050 r.p.m. Cells were pelleted by centrifugation at 3,200 × *g* for 60 min, then the supernatant was discarded. Subsequently, the cells were lysed by a freeze–thaw cycle, followed by resuspension of the dry pellets by vortexing (~1 min) and subsequent addition of 150 µl of lysis buffer (HEPES buffer containing 0.35X BugBuster Protein Extraction Reagent (Novagen) and 0.1% Lysonase Bioprocessing Reagent (Novagen)). The cells were incubated for 20 min at room temperature in lysis buffer on a tabletop shaker (1,000 r.p.m.) and subsequently subjected to 30 min of thermal denaturation at 75 °C. The lysate was cleared by centrifugation for 1 h at 3,200 × *g*, and 60 µl of the supernatant was used for the activity assay. For the reaction, 140 μl of the bait substrate mixture was added to 60-μl aliquots of the cleared lysate in microtitre plates. The assay was carried out in HEPES buffer with a final concentration of 10 µM of the bivalent cation mixture (ZnCl_2_, MnCl_2_ and CaCl_2_) and ~20 µM of the fluorescein phosphate bait substrate mixture. The formation of fluorescein was recorded in a plate reader (Infinite M200; Tecan) for 30–60 min at an excitation wavelength of 480 nm and an emission wavelength of 520 nm.

### NGS and data analysis

After droplet sorting and DNA recovery by electroporation, plasmid DNA was extracted from all obtained *E. coli* colonies using the GeneJET Plasmid Miniprep Kit (ThermoFisher Scientific). The variable region of the library (positions 19–83) was amplified with polymerase chain reaction (PCR) using Q5 polymerase (NEBnext UltraII Q5 Master mix) with primers including adapters for NGS in the overhang and different indices for each sample for multiplexing (Supplementary Table 1). The cycle number was optimized using quantitative PCR with variable template concentrations to be below 15 cycles for the final PCR reaction to minimize amplification bias. AMPure Speed Beads (Beckman Coulter) were used to purify DNA after amplification. The samples were processed into Illumina TruSeq libraries by the University of Cambridge Department of Biochemistry sequencing facility according to the manufacturer’s instructions. Sequencing was performed with one Illumina MiSeq 2 × 300-bp run (20% PhiX spike-in), yielding 8.5 × 10^6^ sequences for the input library, 4.8 × 10^5^ sequences for sorting 1 and 6.5 × 10^5^ sequences for sorting 2. Adapters were removed, reads were merged, and individual sequences were counted using the DiMSum pipeline using the following parameters: cutadaptMinLength 10, vsearchMinQual 20, cutadaptErrorRate 0.6, paired T, indels all, maxSubstitutions 100, mutagenesisType codon, mixedSubstitutions T^85^ giving information on the occurrence of 1.4 million unique variants in the input library versus the libraries at post-sorting. The processed read counts were analysed using custom Python scripts (available at https://github.com/fhlab/Early-evolution)^86^ to count the frequency of truncations and frameshift mutations among all library members and at individual positions.

### Protein expression and purification

A single colony of *E. coli* BL21 carrying the plasmid to express the protein of interest was used to inoculate a starter culture in LB medium and grown at 37 °C for 12 h. This started culture was used to inoculate 1 l of LB medium (1:200 dilution), which was grown at 37 °C until it reached an optical density at 600 nm of 0.6, at which time anhydrotetracycline was added to a final concentration of 200 ng ml^−1^ to induce protein expression. After 12 h of expression at 18 °C, cells were collected via centrifugation (10 min, 4,000 *×* *g*) and stored at −80 °C.

To purify protein, the cells were resuspended in phosphate buffer (50 mM Na_2_HPO_4_, 300 mM NaCl, pH 8.0) and lysed by sonication on ice at 45% amplitude for twenty 10-s bursts with 50 s between sonication events. Following centrifugation (20 min, 18,000 × *g*) and filtration, clarified lysate was loaded onto a nickel column (GE HisTrap HP, 5-ml volume) and purified by imidazole elution (phosphate buffer with 500 mM imidazole). His_6_-tagged protein was eluted with 375 mM imidazole, while untagged protein still weakly stuck to the column and could be eluted with 10 mM imidazole. This was followed by SEC (HiLoad 26/600 Superdex 75 pg) with Tris buffer. The elution time of the peak by SEC corresponds to ~14 kDa when compared to protein standards, which supports a dimeric structure. Mutations did not alter the SEC profile.

To keep the protein reduced throughout, 1 mM dithiothreitol (DTT) was added after elution from the nickel column, and 1 mM tris(2-carboxyethyl)phosphine (TCEP) was added after elution from the sizing column. Proteins were aliquoted, frozen in liquid nitrogen, and stored at −80 °C for subsequent assays. All protein was also purified metal-free (*apo*) by the addition of 5 mM EDTA (pH 8.0) after elution from the nickel column. The EDTA was removed by the subsequent SEC step. For protein oxidation, reducing agent was removed by a PD-10 desalting column (GE Healthcare) exchange to Tris buffer at a final protein concentration of ~120 µM. The protein was then split into samples to which increasing concentrations of hydrogen peroxide were added to promote disulfide formation^87^. The samples were then left in a cold room (4 °C) in the dark for 24 h. In the absence of hydrogen peroxide, the protein remained reduced due to the residual reducing agent, but increasing concentrations of hydrogen peroxide allowed the protein to form disulfide-bonded dimers (Fig. 5c,d). To monitor the presence/formation of disulfide-bonded dimers, proteins were analysed by HPLC as described in the following, or by 12% sodium dodecyl sulfate polyacrylamide gel electrophoresis (SDS–PAGE; Bio-Rad) at 20 µM concentration without added reducing agent in the loading buffer.

### Size-exclusion chromatography

SEC was performed on an ÄKTA Pure FPLC system (General Electric). Preparation-scale SEC was carried out on a HiLoad 26/600 Superdex 75-pg column, and analytical SEC was run on a 5/150GL Superdex 75 increase column (Cytiva). Calibration was done against the external standards γ-globulin (bovine), ovalbumin (chicken), myoglobin (horse) and vitamin B-12 (Bio-Rad).

### Confirmation of protein purity

To assay for contaminating endogenous *E. coli* proteins, both RP-HPLC and MS were used (Supplementary Fig. 7). To validate protein purity by RP-HPLC, 10 µl of purified protein was injected into a C-18 column (Agilent Zorbax 300SB-C18, 5 µM, 2.1 × 150 mm) without dilution or buffer exchange. Solvent A was water with 0.1% trifluoroacetic acid (TFA), and solvent B was acetonitrile with 0.1% TFA. The gradient was 97% solvent A/3% solvent B from 0 to 5 min, 97% solvent A/3% B to 100% solvent B from 5 to 25 min to elute proteins, 100% solvent B from 25 to 30 min to wash the column, and then 97% solvent A/3% solvent B from 30 to 35 min to re-equilibrate. The only peak detected was the de novo protein. The fractions were then run through electrospray ionization MS (ESI-MS; Agilent 6210 TOF LC/MS) to confirm the protein identity. From the HPLC runs, proteins were quantified by their area under the curve (AUC) using the calculated extinction coefficient of 2,980 M^−1^ cm^−1^ for His_6_-tagged proteins and 1,490 M^−1^ cm^−1^ for proteins without His_6_-tags:

1 $${n}={\frac{{\rm{AUC}}\times F}{d\times \varepsilon }}$$

where *n* is the amount of substance of the analyte (in mol), AUC is the area under the curve (in s), *F* is the flow rate (in l s^−1^), *d* is the optical pathlength (in cm) and *ε* is the extinction coefficient (in M^−1^ cm^−1^).

### Steady-state kinetics

Substrate concentrations were chosen to span approximately tenfold below and above *K*_M_, unless limited by substrate solubility. The optimal starting enzyme concentration (*E*_0_) and substrate concentration ranges were determined for each variant and substrate combination by empirical sampling. Kinetics with mini-cAMPase (Table 1) were measured at an enzyme concentration of 50 µM and, because mini-cAMPase functions as a dimer, the kinetic parameters were calculated per protein dimer. The substrates were pre-dissolved in dimethyl sulfoxide (DMSO) at stocks of 200-fold the final concentration to ensure a constant DMSO concentration (0.5%) across all substrate concentrations. Upon measurement, aliquots of these substrate stocks were diluted 1:100 in HEPES buffer containing 5 mM DTT or 1 mM TCEP, of which 100 μl was subsequently mixed with 100 μl of twofold-concentrated enzyme solution in microtitre plate wells. For substrates with a *p*-nitrophenol leaving group, the progress of the reaction was monitored by absorbance at a wavelength of 405 nm in a spectrophotometric microplate reader (Tecan Infinite 200PRO; Tecan) at 25 °C. The initial rates were extracted by a linear fit of the first measurements (at <10% progress of the reaction) for each substrate concentration and normalized with an extinction coefficient determined from a calibration curve. To determine the Michaelis–Menten parameters *k*_cat_ and *K*_M_, the data were fitted to the following equation using the nonlinear fitting function nls() in R^88^:

2 $${\frac{v}{[{E}_{0}]}}={\frac{{k}_{\rm{cat}}\times [S]}{({K}_{\rm{M}}+[S])}}$$

where *v* is the initial rate of the reaction (in mol s^−1^), [*E*_0_] is the initial enzyme concentration (in M), *k*_cat_ is the turnover rate (in s^−1^), *K*_M_ is the Michaelis constant (in M) and [*S*] is the substrate concentration (in M).

For cyclic nucleotide hydrolysis, unless otherwise indicated, the assays used fully reduced proteins in Tris-buffered saline (TBS) and 1 mM TCEP as described above. For each assay, protein aliquots were thawed from storage at −80 °C, diluted to the appropriate concentration, after which metals and substrate were added. Metals and substrate were added from 10X stocks. Nucleotides and metals were in TBS and bis-*p*NPP was in DMSO. All assays included side-by-side samples with substrate, metal and buffer but no protein to measure autohydrolysis, which was used to baseline the measurements.

Cyclic nucleotide hydrolysis was quantified by ultraviolet absorption at 254 nm for AMP and cAMP separated by HPLC, or 260 nm for GMP and cGMP. Assays were performed on an Agilent 1100 series HPLC system with a reverse-phase column (Agilent Zorbax 300SB-C18, 5 μm particle size, 2.1 × 150 mm). Solvent A was water containing 0.1% TFA and solvent B was acetonitrile containing 0.1% TFA. Elution was isocratic, with 3% column B for 5 min, followed by a 5-min flush with solvent B and a 5-min re-equilibration with 3% solvent B. An example separation is shown in Supplementary Fig. 10a. Time-resolved kinetics were followed by 10-µl sample injections using the autosampler module with a 15-min runtime and an isopropanol wash of the needle between injections.

Turnover was quantified by the AUC for AMP or GMP. An external standard curve (Supplementary Fig. 10b) matched the theoretical signal calculated by equation (1), with the NTP extinction coefficient taken from ref. ^89^. The bis-*p*NPP hydrolysis was measured in microtitre plates using the absorbance at 405 nm measured using a Thermo Scientific Varioskan system. Turnover was quantified by an external standard curve using *para*-nitrophenol. dA-P-dA hydrolysis was monitored like the cyclic nucleotides, with turnover calculated using the appearance of both products and the absorbance of the adenine nucleobase present in both products (dA-P and dA). Sample raw data are shown in Supplementary Fig. 12.

### Circular dichroism

CD data were collected on a Chirascan CD spectrometer (Applied Photophysics) from 200 to 260 nm in triplicate and averaged. Far-ultraviolet CD spectra were collected using a 1-mm-pathlength cuvette and 40 μM mini-cAMPase (or alanine mutant) in Tris/HCl buffer or 20 μM S-824 in Tris/HCl buffer.

### NMR

Protein was concentrated with centrifugal filter units (Amicon, 3-kDa molecular weight cutoff) to a final concentration of 1 mg ml^−1^ in PBS. Proton spectra were collected at 25 °C by using a Bruker Avance III 800-MHz spectrometer. The ^1^H chemical shift was referenced to the DOH line.

### Mutagenesis

PCR mutagenesis was performed by whole-plasmid PCR with Q5 high-fidelity polymerase (New England Biolabs) followed by PCR purification (Qiagen) and treatment with KLD Enzyme Mix (New England Biolabs). The primers were made by Sigma-Aldrich and are listed in Supplementary Table 4. His_6_-tagged proteins included a tobacco etch virus protease cleavage recognition site.

### MD simulations

The MD simulations were performed with Amber18^90^: 100-ns simulations were run for 1P68, the 1P68 structure predicted with ESMfold^35^, and the structure of mini-cAMPase dimer predicted by AlphaFold2^32–34^. The system was parametrized using tleap^90^, and enzymes were solvated in a 12.0-Å octahedral box of TIP3P water^91,92^ with net charge neutralized by the addition of sodium ions. The ff19SB force field^93^ was used to describe the protein. All systems were minimized using 10,000 steps of steepest descent followed by 10,000 steps of conjugate gradient. Subsequently, the system was heated from 50 K to 300 K in 20 ps, and then simulated for 100 ns in the NPT ensemble, saving a frame every 100 ps. Langevin dynamics were used with a collision frequency of 0.2 and a 2-fs time step. A Berendsen barostat was used with isotropic position scaling. All bonds involving hydrogens were constrained using the SHAKE algorithm. Ten independent simulations were run per enzyme variant, resulting in a total simulation time of 1.0 µs per variant. All calculations were performed with the Amber18 program package (sander.MPI for minimization and pmemd.cuda for MD simulations)^90^. MD simulations were analysed using CPPTRAJ^94^. All analyses were based on Cα positions. The first 50 ns of each MD run were excluded to allow sufficient time for system equilibration. Root-mean-square deviation (r.m.s.d.) values were calculated compared to the minimized starting structures. Root-mean-square fluctuations (r.m.s.f.) were determined by first calculating an average structure for each replicate, aligning the trajectory against the average structure, and then calculating the r.m.s.f. for each protein residue. Errors indicate the standard error of ten independent replicates.

### Structure prediction

AlphaFold2^32–34^, EMSfold^35^ and MultiSFold^36^ were used to predict the structures of the mini-cAMPase dimer and S-824. AlphaFold2 structure predictions were run with google colab (https://colab.research.google.com/github/sokrypton/ColabFold/blob/main/AlphaFold2.ipynb). EMSfold structure predictions were run for S-824 with https://esmatlas.com/resources. Conformer predictions for S-824 and mini-cAMPase were run with MultiSFold^36^ (http://zhanglab-bioinf.com/MultiSFold).

### Reporting summary

Further information on research design is available in the Nature Portfolio Reporting Summary linked to this article.

## Online content

Any methods, additional references, Nature Portfolio reporting summaries, source data, extended data, supplementary information, acknowledgements, peer review information; details of author contributions and competing interests; and statements of data and code availability are available at 10.1038/s41557-024-01490-4.

### Supplementary information

Supplementary Information Supplementary methods, Figs. 1–13, Tables 1–4, sequences and references.

Reporting Summary

Supplementary Data 1 MD input files, scripts and final structures derived from MD, AlphaFold2, EMSfold and MultiSFold.

Supplementary Data 2 Source data for secondary screening shown in Supplementary Fig. 3.

Supplementary Data 3 Source data for plots in Supplementary Figs. 6–12.

Supplementary Data 4 Source data for Supplementary Fig. 4.

Supplementary Data 5 Source data for Supplementary Fig. 13.

Supplementary Data 6 CAD files of microfluidic chip layouts.

### Source data

Source Data Fig. 2 Data for Fig. 2.

Source Data Fig. 3 Data for Fig. 3a.

Source Data Fig. 4 All data for Fig. 4 plots.

Source Data Fig. 5 All data for Fig. 5 plots and the raw gel. ‘Fig. 5 NMR Data.zip’ is source data for the NMR spectra shown in panel e.

Source Data Fig. 5 All data for Fig. 5 plots and the raw gel. ‘Fig. 5 NMR Data.zip’ is source data for the NMR spectra shown in panel e.

Source Data Fig. 5 All data for Fig. 5 plots and the raw gel. ‘Fig. 5 NMR Data.zip’ is source data for the NMR spectra shown in panel e.

Source Data Extended Data Fig. 1 Data for Extended Data Fig. 1.

Source Data Extended Data Fig. 2 All data for Extended Data Fig. 2 plots.

Source Data Extended Data Fig. 3 All data for Extended Data Fig. 3 plots.

Source Data Extended Data Fig. 4a Statistical source data for Michaelis-Menten plots.

Source Data Extended Data Fig. 4b Statistical source data for Michaelis-Menten plots.

Source Data Extended Data Fig. 4c Statistical source data for Michaelis-Menten plots.

Source Data Extended Data Fig. 4d Statistical source data for Michaelis-Menten plots.

Source Data Extended Data Fig. 5 All data for Extended Data Fig. 5 plots.

Source Data Extended Data Fig. 6 All data for Extended Data Fig. 6 plots and the raw gel.

Source Data Extended Data Fig. 6c All Data for Extended Data Fig. 6 plots and the raw gel.

Source Data Extended Data Fig. 7 Statistical source data for Michaelis-Menten plots.

## Figures and Tables

**Extended Data Table 1:**
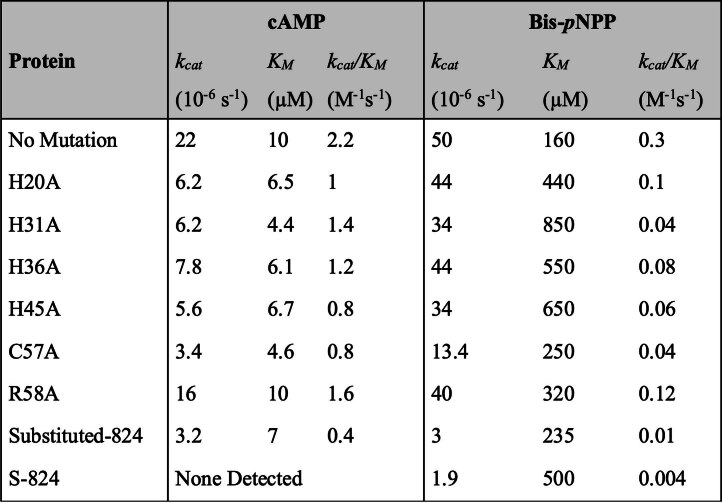
Michaelis-Menten kinetics for alanine scanning mutants of mini-cAMPase

Kinetics were measured at 50 µM enzyme concentration in 50 mM HEPES-NaOH, 150 mM NaCl, 200 µM MnCl_2_, pH 8.0 at 25 °C. Note that for truncated proteins, which form dimers, the kinetics are calculated per protein dimer.

## Data Availability

The gene sequence for mini-cAMPase has been uploaded to GenBank (OQ789719) and is also provided in the Supplementary Information. Next-generation sequencing reads have been uploaded to the European Nucleotide Archive PRJEB66226 (ERP151302). MD input files, scripts and final structures derived from MD, AlphaFold2, EMSfold and MultiSFold are provided in the Supplementary Information. The following publicly available datasets were used for analysis of rate acceleration: 10.1073/pnas.0903951107 (ref. ^80^), 10.1139/v87-315 (ref. ^82^), 10.1073/pnas.0510879103 (ref. ^81^) and 10.1021/ja9733604 (ref. ^95^). CAD files with the microfluidic chip designs are provided as Supplementary Data Files and are also available on https://openwetware.org/wiki/DropBase (ref. ^96^). Further data supporting the main findings of this work are available within the Article, Supplementary Information and source data. Correspondence and requests for materials (for example, plasmid constructs) should be addressed to the correspondence authors. Source data are provided with this paper.

## Code availability

All scripts used for the NGS analysis are available via GitHub at https://github.com/fhlab/Early-evolution (ref. ^86^).

## References

[1] Yeh AH-W (2023). De novo design of luciferases using deep learning. Nature.

[2] Tiessen A, Pérez-Rodríguez P, Delaye-Arredondo LJ (2012). Mathematical modeling and comparison of protein size distribution in different plant, animal, fungal and microbial species reveals a negative correlation between protein size and protein number, thus providing insight into the evolution of proteomes. BMC Res. Notes.

[3] Mandecki W (1990). A method for construction of long randomized open reading frames and polypeptides. Protein Eng..

[4] Prijambada ID (1996). Solubility of artificial proteins with random sequences. FEBS Lett..

[5] Keefe AD, Szostak JW (2001). Functional proteins from a random-sequence library. Nature.

[6] Jensen RA (1976). Enzyme recruitment in evolution of new function. Annu. Rev. Microbiol..

[7] O’Brien PJ, Herschlag D (1999). Catalytic promiscuity and the evolution of new enzymatic activities. Chem. Biol..

[8] Colin P-Y (2015). Ultrahigh-throughput discovery of promiscuous enzymes by picodroplet functional metagenomics. Nat. Commun..

[9] Seelig B, Szostak JW (2007). Selection and evolution of enzymes from a partially randomized non-catalytic scaffold. Nature.

[10] Chao F-A (2013). Structure and dynamics of a primordial catalytic fold generated by in vitro evolution. Nat. Chem. Biol..

[11] Hilvert D (2013). Design of protein catalysts. Annu. Rev. Biochem..

[12] Eck RV, Dayhoff MO (1966). Evolution of the structure of ferredoxin based on living relics of primitive amino acid sequences. Science.

[13] Romero Romero ML, Rabin A, Tawfik DS (2016). Functional proteins from short peptides: Dayhoff’s hypothesis turns 50. Angew. Chem. Int. Ed..

[14] Wei Y, Kim S, Fela D, Baum J, Hecht MH (2003). Solution structure of a de novo protein from a designed combinatorial library. Proc. Natl Acad. Sci. USA.

[15] Wei Y (2003). Stably folded de novo proteins from a designed combinatorial library. Protein Sci..

[16] Ferris JP (1993). Catalysis and prebiotic RNA synthesis. Orig. Life Evol. Biosph..

[17] Bray MS (2018). Multiple prebiotic metals mediate translation. Proc. Natl Acad. Sci. USA.

[18] Muchowska KB (2017). Metals promote sequences of the reverse Krebs cycle. Nat. Ecol. Evol..

[19] Kamtekar S, Schiffer JM, Xiong H, Babik JM, Hecht MH (1993). Protein design by binary patterning of polar and nonpolar amino acids. Science.

[20] Karas C, Hecht M (2020). A strategy for combinatorial cavity design in de novo proteins. Life.

[21] Colin P-Y, Zinchenko A, Hollfelder F (2015). Enzyme engineering in biomimetic compartments. Curr. Opin. Struct. Biol..

[22] Gantz M, Aleku GA, Hollfelder F (2022). Ultrahigh-throughput screening in microfluidic droplets: a faster route to new enzymes. Trends Biochem. Sci..

[23] Baret J-C (2009). Fluorescence-activated droplet sorting (FADS): efficient microfluidic cell sorting based on enzymatic activity. Lab Chip.

[24] Fowler DM (2010). High-resolution mapping of protein sequence-function relationships. Nat. Methods.

[25] Hietpas RT, Jensen JD, Bolon DNA (2011). Experimental illumination of a fitness landscape. Proc. Natl Acad. Sci. USA.

[26] Larsen AC (2016). A general strategy for expanding polymerase function by droplet microfluidics. Nat. Commun..

[27] Check Hayden E (2008). Chemistry: designer debacle. Nature.

[28] O’Brien PJ, Herschlag D (2001). Functional interrelationships in the alkaline phosphatase superfamily: phosphodiesterase activity of *Escherichia coli* alkaline phosphatase. Biochemistry.

[29] Nielsen LD, Monard D, Rickenberg HV (1973). Cyclic 3′,5′-adenosine monophosphate phosphodiesterase of *Escherichia coli*. J. Bacteriol..

[30] Imamura R (1996). Identification of the cpdA gene encoding cyclic 3ʹ,5ʹ-adenosine monophosphate phosphodiesterase in *Escherichia coli*. J. Biol. Chem..

[31] Schwer B, Khalid F, Shuman S (2016). Mechanistic insights into the manganese-dependent phosphodiesterase activity of yeast Dbr1 with bis-*p*-nitrophenylphosphate and branched RNA substrates. RNA.

[32] Jumper J (2021). Highly accurate protein structure prediction with AlphaFold. Nature.

[33] Evans, R. et al. Protein complex prediction with AlphaFold-Multimer. Preprint at *bioRxiv*10.1101/2021.10.04.463034 (2022).

[34] Mirdita M (2022). ColabFold: making protein folding accessible to all. Nat. Methods.

[35] Lin Z (2023). Evolutionary-scale prediction of atomic-level protein structure with a language model. Science.

[36] Hou, M. et al. Protein multiple conformation prediction using multi-objective evolution algorithm. *Interdiscip. Sci. Comput. Life Sci.*10.1007/s12539-023-00597-5 (2024). 10.1007/s12539-023-00597-5 38190097

[37] Bar-Even A (2011). The moderately efficient enzyme: evolutionary and physicochemical trends shaping enzyme parameters. Biochemistry.

[38] Copley SD, Newton MS, Widney KA (2023). How to recruit a promiscuous enzyme to serve a new function. Biochemistry.

[39] Radzicka A, Wolfenden R (1995). A proficient enzyme. Science.

[40] Emond S (2020). Accessing unexplored regions of sequence space in directed enzyme evolution via insertion/deletion mutagenesis. Nat. Commun..

[41] Miton CM, Tokuriki N (2023). Insertions and deletions (indels): a missing piece of the protein engineering jigsaw. Biochemistry.

[42] Savino S, Desmet T, Franceus J (2022). Insertions and deletions in protein evolution and engineering. Biotechnol. Adv..

[43] Wang MS, Hecht MH (2020). A completely de novo ATPase from combinatorial protein design. J. Am. Chem. Soc..

[44] Kurihara K (2023). Crystal structure and activity of a de novo enzyme, ferric enterobactin esterase Syn-F4. Proc. Natl Acad. Sci. USA.

[45] Donnelly AE, Murphy GS, Digianantonio KM, Hecht MH (2018). A de novo enzyme catalyzes a life-sustaining reaction in *Escherichia coli*. Nat. Chem. Biol..

[46] Lombardi A, Pirro F, Maglio O, Chino M, DeGrado WF (2019). De novo design of four-helix bundle metalloproteins: one scaffold, diverse reactivities. Acc. Chem. Res..

[47] Chalkley MJ, Mann SI, DeGrado WF (2022). De novo metalloprotein design. Nat. Rev. Chem..

[48] Studer S (2018). Evolution of a highly active and enantiospecific metalloenzyme from short peptides. Science.

[49] Basler S (2021). Efficient Lewis acid catalysis of an abiological reaction in a de novo protein scaffold. Nat. Chem..

[50] Razkin J, Lindgren J, Nilsson H, Baltzer L (2008). Enhanced complexity and catalytic efficiency in the hydrolysis of phosphate diesters by rationally designed helix-loop-helix motifs. ChemBioChem.

[51] Chen J (2005). An asymmetric dizinc phosphodiesterase model with phenolate and carboxylate bridges. Inorg. Chem..

[52] Matange N (2015). Revisiting bacterial cyclic nucleotide phosphodiesterases: cyclic AMP hydrolysis and beyond. FEMS Microbiol. Lett..

[53] Agresti JJ (2010). Ultrahigh-throughput screening in drop-based microfluidics for directed evolution. Proc. Natl Acad. Sci. USA.

[54] Kintses B (2012). Picoliter cell lysate assays in microfluidic droplet compartments for directed enzyme evolution. Chem. Biol..

[55] Obexer R (2017). Emergence of a catalytic tetrad during evolution of a highly active artificial aldolase. Nat. Chem..

[56] Ma F (2018). Efficient molecular evolution to generate enantioselective enzymes using a dual-channel microfluidic droplet screening platform. Nat. Commun..

[57] Debon A (2019). Ultrahigh-throughput screening enables efficient single-round oxidase remodelling. Nat. Catal..

[58] Neun S (2022). Functional metagenomic screening identifies an unexpected β-glucuronidase. Nat. Chem. Biol..

[59] Schnettler JD, Klein OJ, Kaminski TS, Colin P-Y, Hollfelder F (2023). Ultrahigh-throughput directed evolution of a metal-free α/β-hydrolase with a Cys-His-Asp triad into an efficient phosphotriesterase. J. Am. Chem. Soc..

[60] Gantz M, Neun S, Medcalf EJ, van Vliet LD, Hollfelder F (2023). Ultrahigh-throughput enzyme engineering and discovery in in vitro compartments. Chem. Rev..

[61] Gielen F (2016). Ultrahigh-throughput–directed enzyme evolution by absorbance-activated droplet sorting (AADS). Proc. Natl Acad. Sci. USA.

[62] Holland-Moritz DA (2020). Mass Activated Droplet Sorting (MADS) enables high-throughput screening of enzymatic reactions at nanoliter scale. Angew. Chem..

[63] Seelig B (2011). mRNA display for the selection and evolution of enzymes from in vitro-translated protein libraries. Nat. Protoc..

[64] Lee J, Blaber M (2011). Experimental support for the evolution of symmetric protein architecture from a simple peptide motif. Proc. Natl Acad. Sci. USA.

[65] Johnston IG (2022). Symmetry and simplicity spontaneously emerge from the algorithmic nature of evolution. Proc. Natl Acad. Sci. USA.

[66] Alvarez-Carreño C, Gupta RJ, Petrov AS, Williams LD (2022). Creative destruction: new protein folds from old. Proc. Natl Acad. Sci. USA.

[67] Ma B, Nussinov R (2010). Enzyme dynamics point to stepwise conformational selection in catalysis. Curr. Opin. Chem. Biol..

[68] Nashine VC, Hammes-Schiffer S, Benkovic SJ (2010). Coupled motions in enzyme catalysis. Curr. Opin. Chem. Biol..

[69] Kern D (2021). From structure to mechanism: skiing the energy landscape. Nat. Methods.

[70] Tokuriki N, Tawfik DS (2009). Protein dynamism and evolvability. Science.

[71] Campbell E (2016). The role of protein dynamics in the evolution of new enzyme function. Nat. Chem. Biol..

[72] Vamvaca K, Vögeli B, Kast P, Pervushin K, Hilvert D (2004). An enzymatic molten globule: efficient coupling of folding and catalysis. Proc. Natl Acad. Sci. USA.

[73] Dellus-Gur E (2015). Negative epistasis and evolvability in TEM-1 β-lactamase—the thin line between an enzyme’s conformational freedom and disorder. J. Mol. Biol..

[74] Mabbitt PD (2016). Conformational disorganization within the active site of a recently evolved organophosphate hydrolase limits its catalytic efficiency. Biochemistry.

[75] Smith BA, Mularz AE, Hecht MH (2015). Divergent evolution of a bifunctional de novo protein. Protein Sci. Publ. Protein Soc..

[76] Bershtein S, Segal M, Bekerman R, Tokuriki N, Tawfik DS (2006). Robustness–epistasis link shapes the fitness landscape of a randomly drifting protein. Nature.

[77] Yang G (2019). Higher-order epistasis shapes the fitness landscape of a xenobiotic-degrading enzyme. Nat. Chem. Biol..

[78] Kaltenbach M, Jackson CJ, Campbell EC, Hollfelder F, Tokuriki N (2015). Reverse evolution leads to genotypic incompatibility despite functional and active site convergence. eLife.

[79] Park Y, Metzger BPH, Thornton JW (2022). Epistatic drift causes gradual decay of predictability in protein evolution. Science.

[80] van Loo B (2010). An efficient, multiply promiscuous hydrolase in the alkaline phosphatase superfamily. Proc. Natl Acad. Sci. USA.

[81] Schroeder GK, Lad C, Wyman P, Williams NH, Wolfenden R (2006). The time required for water attack at the phosphorus atom of simple phosphodiesters and of DNA. Proc. Natl Acad. Sci. USA.

[82] Chin J, Zou X (1987). Catalytic hydrolysis of cAMP. Can. J. Chem..

[83] Skerra A (1994). Use of the tetracycline promoter for the tightly regulated production of a murine antibody fragment in *Escherichia coli*. Gene.

[84] Neun, S., Kaminski, T. S. & Hollfelder, F. in *Methods in Enzymology* Vol. 628 (eds Allbritton, N. L. & Kovarik, M. L.) 95–112 (Academic Press, 2019).

[85] Faure AJ, Schmiedel JM, Baeza-Centurion P, Lehner B (2020). DiMSum: an error model and pipeline for analyzing deep mutational scanning data and diagnosing common experimental pathologies. Genome Biol..

[86] Hollfelder, F. et al. Early-evolution. *GitHub* https://github.com/fhlab/Early-evolution (2023).

[87] Rehder DS, Borges CR (2010). Cysteine sulfenic acid as an intermediate in disulfide bond formation and nonenzymatic protein folding. Biochemistry.

[88] R Core Team. *R*: *A Language and Environment for Statistical Computing* (R Foundation for Statistical Computing, 2017).

[89] Cavaluzzi MJ, Borer PN (2004). Revised UV extinction coefficients for nucleoside‐5′‐monophosphates and unpaired DNA and RNA. Nucleic Acids Res..

[90] Case, D. A. et al. Amber v.2018 (Univ. California, San Francisco, 2018).

[91] Le Grand S, Götz AW, Walker RC (2013). SPFP: speed without compromise—a mixed precision model for GPU accelerated molecular dynamics simulations. Comput. Phys. Commun..

[92] Salomon-Ferrer R, Götz AW, Poole D, Le Grand S, Walker RC (2013). Routine microsecond molecular dynamics simulations with AMBER on GPUs. 2. Explicit solvent particle Mesh Ewald. J. Chem. Theory Comput..

[93] Tian C (2020). ff19SB: amino-acid-specific protein backbone parameters trained against quantum mechanics energy surfaces in solution. J. Chem. Theory Comput..

[94] Roe DR, Cheatham TEI (2013). PTRAJ and CPPTRAJ: software for processing and analysis of molecular dynamics trajectory data. J. Chem. Theory Comput..

[95] Wolfenden R, Ridgway C, Young G (1998). Spontaneous hydrolysis of ionized phosphate monoesters and diesters and the proficiencies of phosphatases and phosphodiesterases as catalysts. J. Am. Chem. Soc..

[96] Hollfelder, F. et al. DropBase. *OpenWetWare* https://openwetware.org/wiki/DropBase (2023).

