# Supplementary material for: Selection of a promiscuous minimalist cAMP phosphodiesterase from a library of de novo designed proteins
Source: Nat Chem. 2024 May 3;16(7):1200–8. doi: 10.1038/s41557-024-01490-4 (PMC11230910; doi:10.1038/s41557-024-01490-4)
Supplement: Supplementary file 2 — Reporting Summary [file 41557_2024_1490_MOESM2_ESM.pdf]

nature portfolio

Corresponding author(s): Florian Hollfelder & Michael Hecht

Last updated by author(s): 12/02/2024

Reporting Summary

Nature Portfolio wishes to improve the reproducibility of the work that we publish. This form provides structure for consistency and transparency in reporting. For further information on Nature Portfolio policies, see our Editorial Policies and the Editorial Policy Checklist.

Statistics

For all statistical analyses, confirm that the following items are present in the figure legend, table legend, main text, or Methods section.

n/a	Confirmed	
☐	☒	The exact sample size (*n*) for each experimental group/condition, given as a discrete number and unit of measurement
☐	☒	A statement on whether measurements were taken from distinct samples or whether the same sample was measured repeatedly
☒	☐	The statistical test(s) used AND whether they are one- or two-sided<br>*Only common tests should be described solely by name; describe more complex techniques in the Methods section.*
☒	☐	A description of all covariates tested
☒	☐	A description of any assumptions or corrections, such as tests of normality and adjustment for multiple comparisons
☐	☒	A full description of the statistical parameters including central tendency (e.g. means) or other basic estimates (e.g. regression coefficient) AND variation (e.g. standard deviation) or associated estimates of uncertainty (e.g. confidence intervals)
☒	☐	For null hypothesis testing, the test statistic (e.g. *F*, *t*, *r*) with confidence intervals, effect sizes, degrees of freedom and *P* value noted<br>*Give P values as exact values whenever suitable.*
☒	☐	For Bayesian analysis, information on the choice of priors and Markov chain Monte Carlo settings
☒	☐	For hierarchical and complex designs, identification of the appropriate level for tests and full reporting of outcomes
☒	☐	Estimates of effect sizes (e.g. Cohen's *d*, Pearson's *r*), indicating how they were calculated

Our web collection on statistics for biologists contains articles on many of the points above.

Software and code

Policy information about availability of computer code

Data collection	Absorbance and Fluorescence Data with Tecan Infinite 200 PRO plate reader: Tecan i-control 1.10 and Spectramax iD5 (Molecular Devices): SoftMax Pro 7.0.3; droplet fluorescence measurements were carried out in a custom-built droplet sorter with a peak detection algorithm (LabView 8.2, National Instruments) described in Colin et al. (2015) Nat Commun, doi: 10.1038/ncomms10008; Next generation sequencing was carried out using Illumina MiSeq (2 x 300 bp) technology. HPLC reaction kinetics: Agilent 1100 with a reverse phase column (Agilent Zorbax 300SB-C18, 5 µM, 2.1 × 150 mm). Mass Spectra: ESI-MS (Agilent 6210 TOF LC/MS) in positive mode. NMR: Bruker Avance III 800 MHz spectrometer and analyzed with MestReNova. Circular Dichroism: Chirascan CD spectrometer from Applied Photophysics. MD simulations: Amber18. Structure prediction: AlphaFold2, EMSfold, and MultiSFold.
Data analysis	The following software was used in this study: Pymol 2.4.2, ColabFold (https://github.com/sokrypton/ColabFold), python 3.6.11 and 3.7, DiMSum https://github.com/lehner-lab/DiMSum, Microsoft Excel v16.71, R Statistical Software v3.4.3, MassHunter Workstation Plus 11.0, custom python scripts: https://github.com/fhlab/Early-evolution

For manuscripts utilizing custom algorithms or software that are central to the research but not yet described in published literature, software must be made available to editors and reviewers. We strongly encourage code deposition in a community repository (e.g. GitHub). See the Nature Portfolio guidelines for submitting code & software for further information.

Data

Policy information about availability of data

All manuscripts must include a data availability statement. This statement should provide the following information, where applicable:

- Accession codes, unique identifiers, or web links for publicly available datasets
- A description of any restrictions on data availability
- For clinical datasets or third party data, please ensure that the statement adheres to our policy

The gene sequence of mini-cAMPase has been uploaded to GenBank (OQ789719) and is also provided in the Supporting Information. Next-generation sequencing reads have been uploaded to the European Nucleotide Archive PRJEB66226 (ERP151302). MD input files, scripts, and final structures derived from MD, AlphaFold2, EMSfold, and MultiSFold are provided in the Supplementary Material.
The following publicly available datasets were used for analysis of rate acceleration: https://doi.org/10.1073/pnas.0903951107, https://doi.org/10.1139/v87-315, https://doi.org/10.1073/pnas.0510879103,https://doi.org/10.1021/ja9733604.
CAD files with the microfluidic chip designs are supplied in the Supplementary Data and are also available on https://openwetware.org/wiki/DropBase.
Further data supporting the main findings of this work are available within the Article, Supplementary Information and provided source data. Correspondence and requests for material (e.g., plasmid constructs) should be addressed to M.H.H. (hecht@princeton.edu) or F.H. (fh111@cam.ac.uk).

Field-specific reporting

Please select the one below that is the best fit for your research. If you are not sure, read the appropriate sections before making your selection.

☒ Life sciences ☐ Behavioural & social sciences ☐ Ecological, evolutionary & environmental sciences

For a reference copy of the document with all sections, see nature.com/documents/nr-reporting-summary-flat.pdf

Life sciences study design

All studies must disclose on these points even when the disclosure is negative.

Sample size	Sample sizes for kinetic analysis (i.e. the number of data points describing initial rates in a Michaelis-Menten curve) were chosen to ensure that the non-linear curve could be reliably fit. (In cases where solubility precluded coverage of a saturation profile, this was explicitly pointed out, highlighting that the actual error could be larger than indicated.)<br>In the droplet-based screen 10.3 million emulsion droplets were screened, so that a theoretical 2.6-fold library overscreening was achieved, meaning every library member was theoretically experimentally tested 2.6 times in an occupied droplet (1.7 times in a single occupied droplet). This sample size was calculated based on the library size (1.7 million) and assuming Poisson distribution (P(k)= (λk *e-λ)/k!) with λ = 0.43. The screen was repeated on the top ≈ 0.5 % brightest clones with a 8.4-fold library overscreening (47 000 clones, 4.4 million droplets screened, λ = 0.1).
Data exclusions	No data were excluded from the studies.
Replication	Michaelis-Menten parameters were derived from multiple datapoints that collectively also suggest excellent reproducibility. All Michaelis-Menten kinetics were performed at least twice independently. All attempts to replicate were successful.
Randomization	Randomization was not relevant to this study as samples were not grouped for experiments or analyses.
Blinding	Blinding was not relevant to this study as samples were not grouped for experiments or analyses.

Reporting for specific materials, systems and methods

We require information from authors about some types of materials, experimental systems and methods used in many studies. Here, indicate whether each material, system or method listed is relevant to your study. If you are not sure if a list item applies to your research, read the appropriate section before selecting a response.

Materials & experimental systems

n/a	Involved in the study	
☒	☐	Antibodies
☒	☐	Eukaryotic cell lines
☒	☐	Palaeontology and archaeology
☒	☐	Animals and other organisms
☒	☐	Human research participants
☒	☐	Clinical data
☒	☐	Dual use research of concern

Methods

n/a	Involved in the study	
☒	☐	ChIP-seq
☒	☐	Flow cytometry
☒	☐	MRI-based neuroimaging
