# Supplementary figures and images for: Selection of a promiscuous minimalist cAMP phosphodiesterase from a library of de novo designed proteins
Source: Nat Chem. 2024 May 3;16(7):1200–8. doi: 10.1038/s41557-024-01490-4 (PMC11230910; doi:10.1038/s41557-024-01490-4)

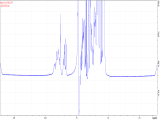

Supplement: Supplementary file 12 — All data for Fig. 5 plots and the raw gel. ‘Fig. 5 NMR Data.zip’ is source data for the NMR spectra shown in panel e. [file 41557_2024_1490_MOESM12_ESM.zip › NMR Data/miniCAMPase_reduced/11/pdata/1/thumb.png]

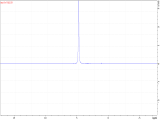

Supplement: Supplementary file 12 — All data for Fig. 5 plots and the raw gel. ‘Fig. 5 NMR Data.zip’ is source data for the NMR spectra shown in panel e. [file 41557_2024_1490_MOESM12_ESM.zip › NMR Data/miniCAMPase_reduced/10/pdata/1/thumb.png]

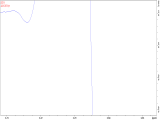

Supplement: Supplementary file 12 — All data for Fig. 5 plots and the raw gel. ‘Fig. 5 NMR Data.zip’ is source data for the NMR spectra shown in panel e. [file 41557_2024_1490_MOESM12_ESM.zip › NMR Data/S824/11/pdata/1/thumb.png]

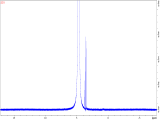

Supplement: Supplementary file 12 — All data for Fig. 5 plots and the raw gel. ‘Fig. 5 NMR Data.zip’ is source data for the NMR spectra shown in panel e. [file 41557_2024_1490_MOESM12_ESM.zip › NMR Data/S824/10/pdata/1/thumb.png]

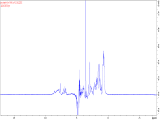

Supplement: Supplementary file 12 — All data for Fig. 5 plots and the raw gel. ‘Fig. 5 NMR Data.zip’ is source data for the NMR spectra shown in panel e. [file 41557_2024_1490_MOESM12_ESM.zip › NMR Data/miniCAMPase_oxidised/11/pdata/1/thumb.png]

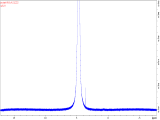

Supplement: Supplementary file 12 — All data for Fig. 5 plots and the raw gel. ‘Fig. 5 NMR Data.zip’ is source data for the NMR spectra shown in panel e. [file 41557_2024_1490_MOESM12_ESM.zip › NMR Data/miniCAMPase_oxidised/10/pdata/1/thumb.png]

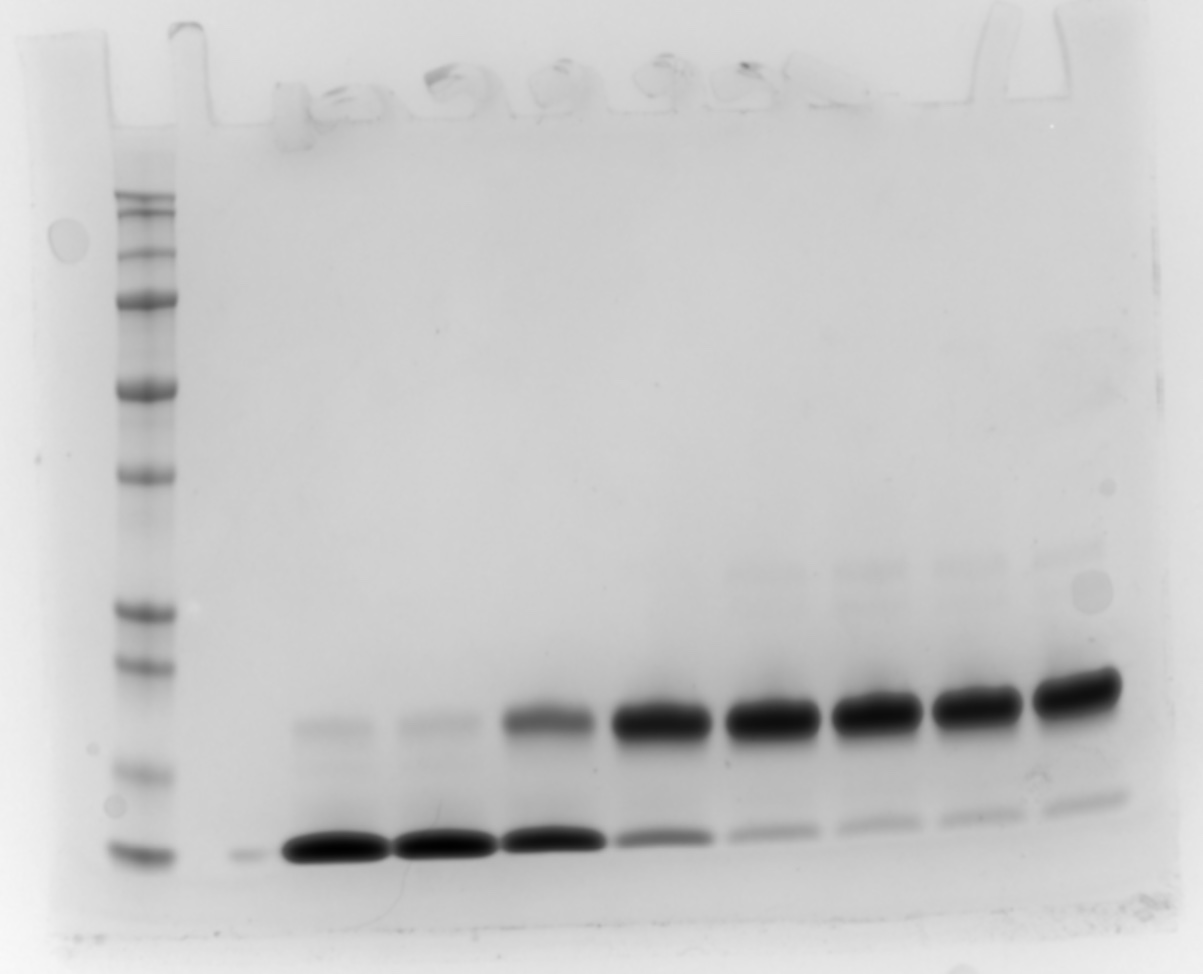

Supplement: Supplementary file 14 — All data for Fig. 5 plots and the raw gel. ‘Fig. 5 NMR Data.zip’ is source data for the NMR spectra shown in panel e. [file 41557_2024_1490_MOESM14_ESM.tif]

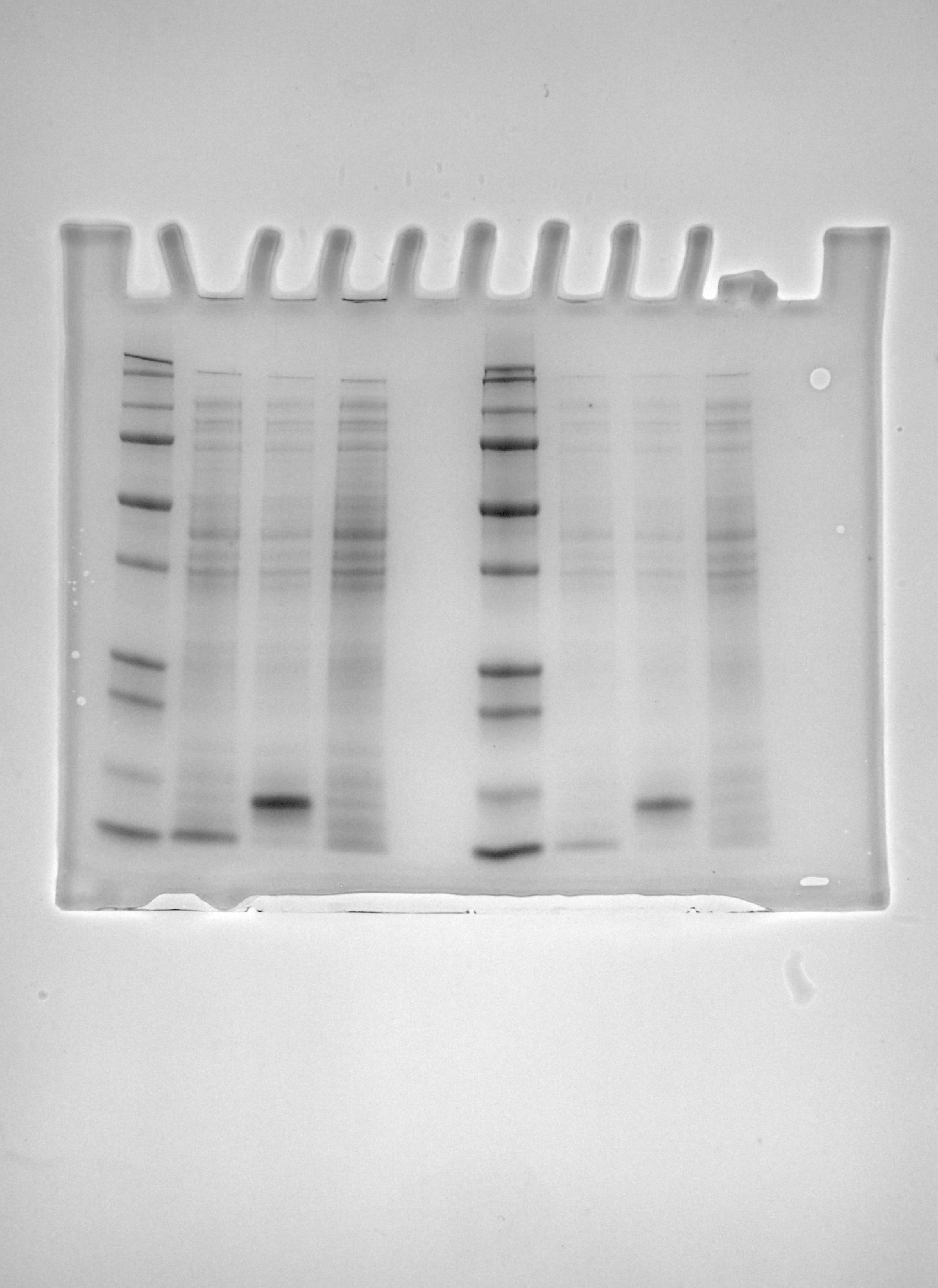

Supplement: Supplementary file 24 — All Data for Extended Data Fig. 6 plots and the raw gel. [file 41557_2024_1490_MOESM24_ESM.jpg]
